# Supplementary material for: Nuclear export is a limiting factor in eukaryotic mRNA metabolism
Source: PLoS Comput Biol. 2024 May 16;20(5):e1012059. doi: 10.1371/journal.pcbi.1012059 (PMC11135743; doi:10.1371/journal.pcbi.1012059)
Supplement: S1 Appendix — (PDF) [file pcbi.1012059.s001.pdf]

S1 Appendix

1 Experimental validation of subcellular fractionation and 4sU conversion

The purity of the fractions was assessed by western blots with GAPDH and α-tubulin for the cytosolic fraction and with RNA Pol II CTD - Ser2P, U1snRNP70 and Histone H3 for the nuclear fraction. According to the Western blots, there was no significant cross-contamination of the individual fractions.

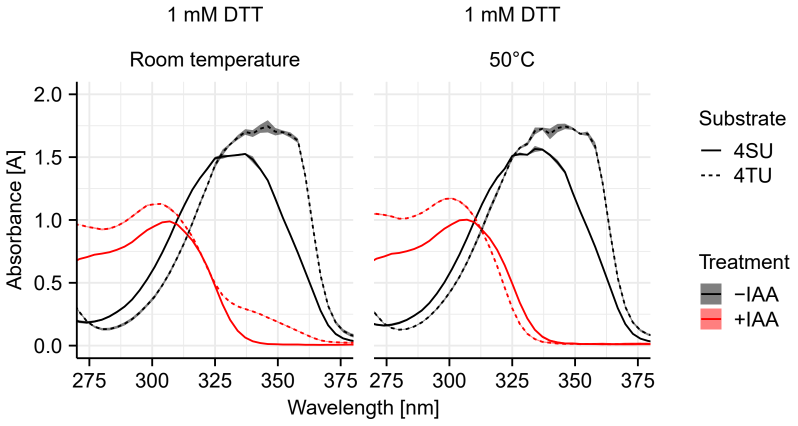

Fig A. **Absorption spectra of 4-thiouracil (4TU) and 4-thiouridine (4SU) before and after treatment with iodoacetamide (IAA) showing the shift in the absorption maxima of reactant and product.** Data represent mean absorbance (line) ± s.d. (ribbon) of independent experiments (n = 3) comparing the effect of temperature on conversion efficiency in presence of 1 mM DTT.

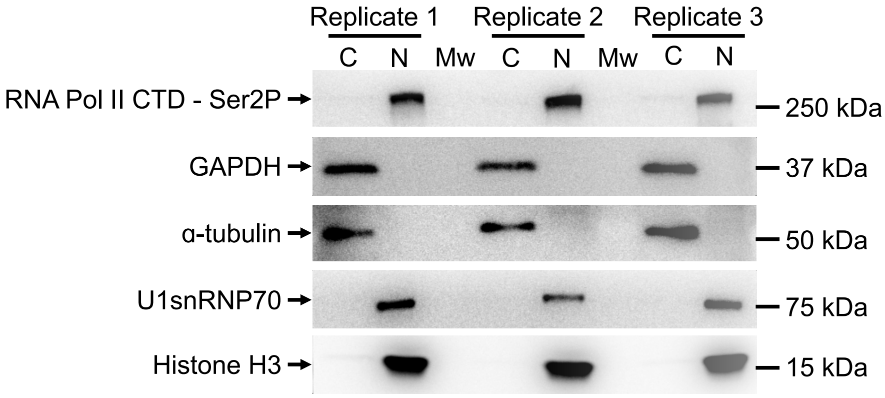

Fig B. **Validation of subcellular fractionation.** Shown are three replicate measures of Western Blots using GAPDH and α-tubulin for the cytosolic fraction, as well as RNA Pol II CTD - Ser2P, U1snRNP70 and Histone H3 for the nuclear fraction.

The conversion efficiency of 4sU was evaluated by absorption spectra before and after treatment with IAA.

2 Basic read alignment

Reads were mapped to human reference genome hg19 with slamdunk [1] (version 0.3.0, settings: -n 100, -m) (Fig C, Fig D, Fig E). We chose slamdunk after comparison with bowtie2 [2] (version 2.2.6, default settings) and hisat2 [3] (version 2.0.0-beta, settings: -mp 1,1 , --no-spliced-alignment) (Fig C).

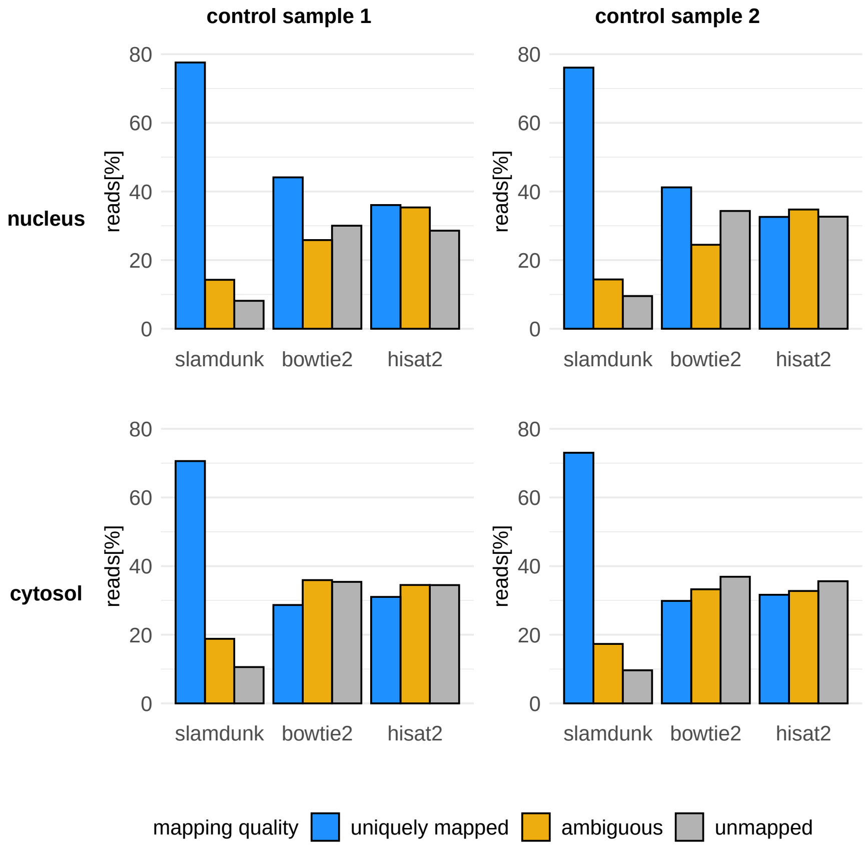

Fig C. **Mapping statistics of the control data sets, shown for both control samples and the nuclear and cytosolic fraction.** Uniquely mapped reads: reads with exactly one alignment (bowtie2 and hisat2), or reads with exactly one reliable alignment (slamdunk). Ambiguous reads: reads with more than one alignment (bowtie2 and hisat2), or reads with more than one reliable alignment or with only unreliable alignments (slamdunk). Unmapped reads: reads with no alignment (slamdunk, bowtie2, hisat2).

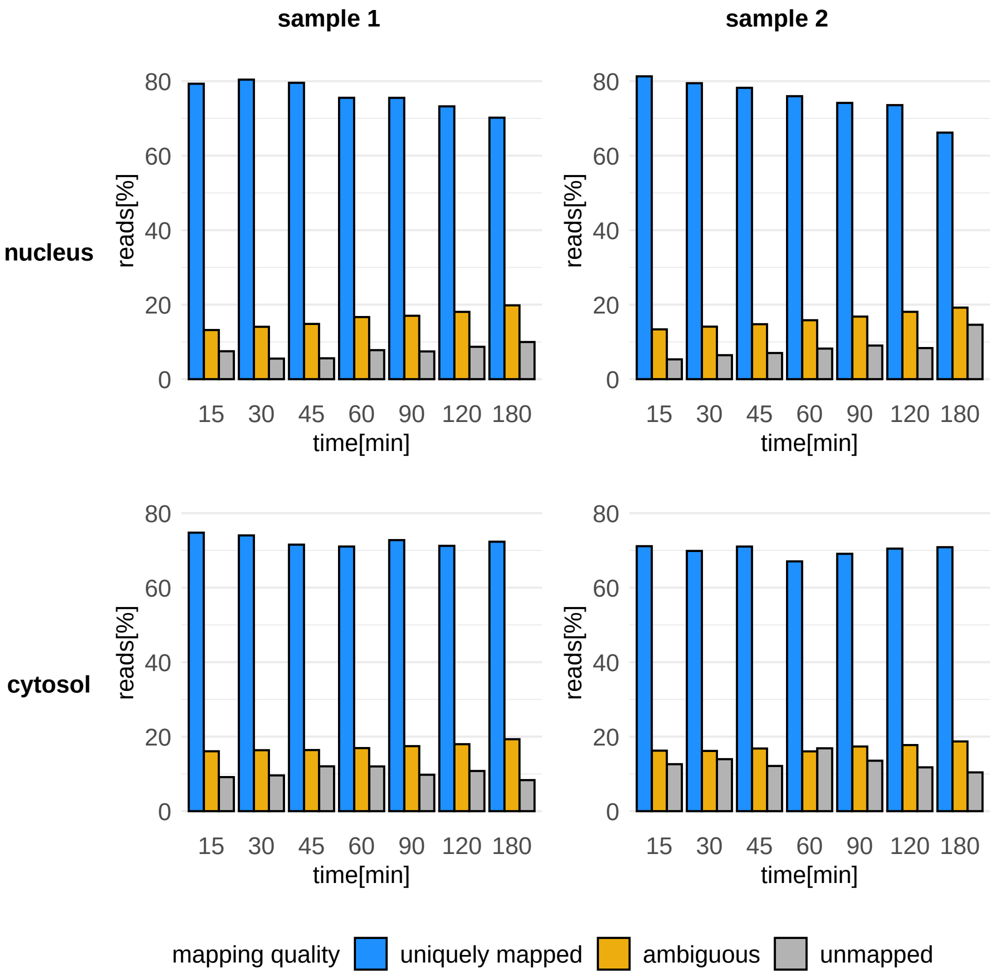

Fig D. **Mapping statistics for both time series and the nuclear and cytosolic fraction.** Uniquely mapped reads: reads with exactly one reliable alignment. Ambiguous reads: reads with more than one reliable alignment or with only unreliable alignments. Unmapped reads: reads with no alignment.

3 Assignment of reads to 3'UTRs and annotation of 3'UTRs

The BED file defining hg19 annotated 3'UTRs was downloaded from UCSC [4]. Overlapping 3'UTRs were merged, considering strand orientation. Uniquely mapped reads were assigned to a specific 3'UTR if their alignment overlapped with the UTR with at least one nucleotide position. Mapping orientation was taken into account during read assignment, as reads derived from (-) strand genomic regions map forwards and reads derived from (+) strand genomic regions map reversely as a consequence of the experimental setup (Fig E). To annotate 3'UTRs with their corresponding genes, the hg19 genome annotation was downloaded from NCBI RefSeq [5] (S4 Table). A 3'UTR region was assigned to a gene if it overlapped with at least one

nucleotide position. The same procedure was applied to all non-3'UTR peaks.

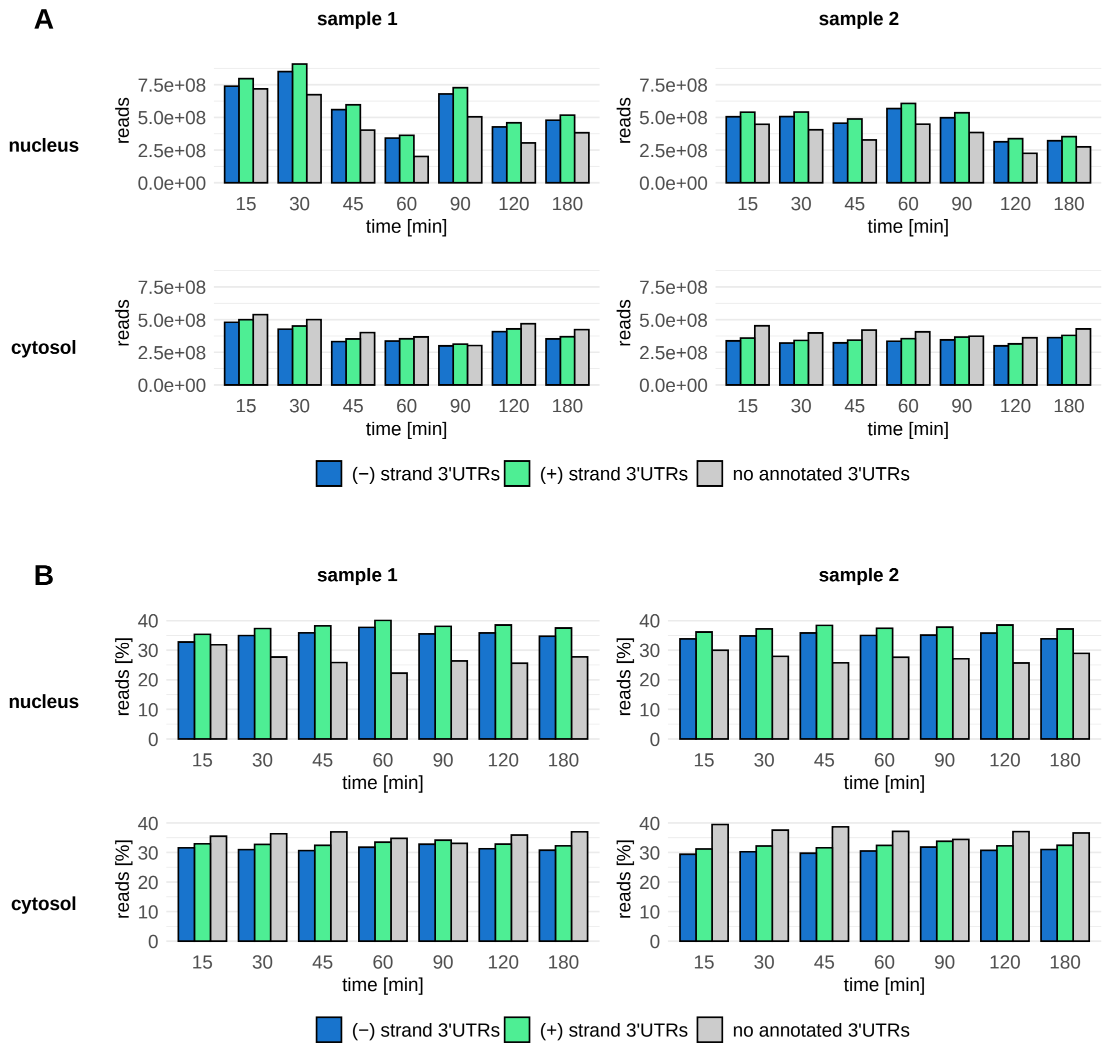

Fig E. **Assignment of uniquely mapped reads of the SLAM-seq labeling samples to hg19 annotated 3'UTRs.** Overlapping 3'UTRs were previously merged considering strand orientation. (A) Absolute read counts. (B) Relative read counts.

4 Peak calling

To identify 3' transcript isoforms and transcripts with internal priming sites, we searched the genome for positions with a particularly high coverage of 3' ends of mapped reads ("peaks", to be defined below). By assigning only count to the 3' end position of a mapped read, we generated coverage profiles for the nuclear and

cytosolic fractions of every measurement time point and each of the two replicate time series. Due biological variation and in the priming of the reverse transcription, the 3' poly-A site of a transcript might not be determined up to single nucleotide resolution. To obtain an (unbiased) idea on how spatially constrained such poly-A sites are in practice, we first binarized the coverage profiles in a ±1000bp window around each genomic position (0 = no count, 1 = at least one count at a given nucleotide position) and then averaged these profiles across all genomic positions that were covered at least once (Fig F and Fig G). The shape of these profiles indicated that counts typically aggregate in a ±50bp region around a central position, the closer the more.We therefore decided to define peak centers as the local coverage maxima of the genome-wide coverage profile that has been convolved with a triangular smoothing kernel of with ±50 (i.e., the function $f(x) = \max(1 - |x|/51, 0)$) (Fig F and Fig G). Peaks or peak regions are then the regions spanning -50bp to +50bp around a peak center.We identified a total of 98,102 peaks, 15,125 of which were measured with at least one count in each time series, time point and compartment, and 1,262,263 peaks for the reads not assigned to annotated 3'UTRs, 4481 of which were measured with at least one count in each time series, time point and compartment.

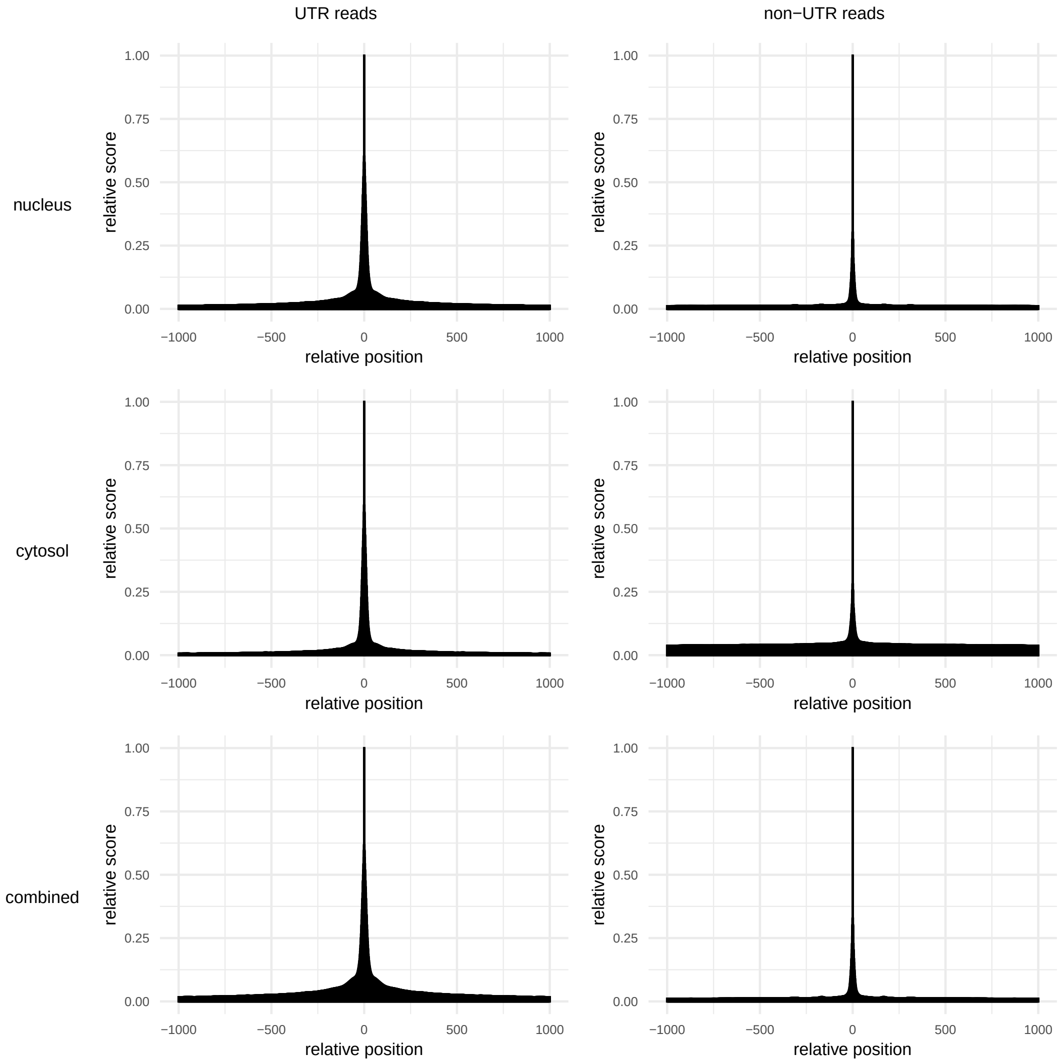

Fig F. **Relative coverage profiles.** Profiles were created separately for the reads assigned to annotated 3'UTRs (UTR-reads) and reads not assigned to such (non-UTR reads) as well as the nuclear and cytosolic fraction. The combined profile is based on the coverage of the nuclear and cyosolic fraction added up.

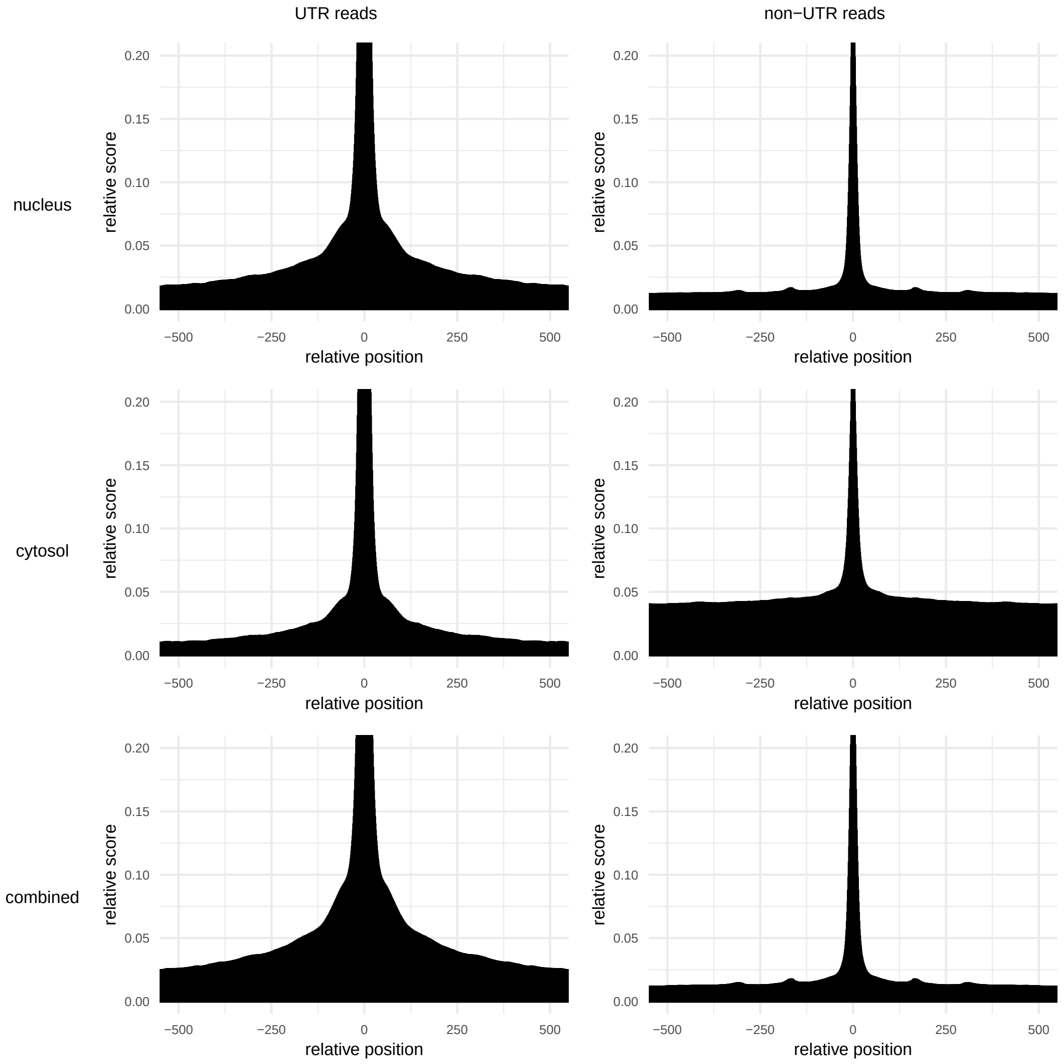

Fig G. **Relative coverage profiles as shown in Fig F with trimmed y-axis for better visualization.**

5 Estimation of the labeling efficiency and new/total ratios

Recall the notation from the Methods. Fix a 3'UTR respectively a peak region onto which J reads were mapped. Given a read $j = 1, ..., J$, let T_j be its number of T-positions in the genomic sequence of the read alignment, and let $o_j \in 0, 1, ..., T_j$ be the number of positions at which we observe T>C transitions. Let $h_j \in \{0, 1\}$ be a hidden variable indicating whether read j originates from a pre-existing RNA ($h_j = 0$), or a newly synthesized RNA ($h_j = 1$). Let $\rho \in [0, 1]$ be the proportion of newly synthesized RNAs in the RNA population from which read j was drawn. Let $\ell \in [0, 1]$ be the labeling efficiency, i.e., the probability by which a T>C conversion happens in a newly synthesized RNA. Let $p_0 \in [0, 1]$ be the probability of a T>C

sequencing error. Let $\epsilon \in [0, 1]$ be the probability of a C>non-C sequencing error, and denote by

$$p_1 = \ell(1-\epsilon) + (1-\ell)p_0 = \ell(1-\epsilon-p_0) + p_0 \tag{1}$$

the probability of observing a T>C conversion in a newly synthesized read. Denote the parameters by $\Theta = (\rho, \ell, p_0, \epsilon)$.

To estimate sequencing error rates p_0 and ϵ, we calculated the single-nucleotide mismatch rates for the nuclear and the cytosolic compartment in the control samples. The T>C probability p_0 was estimated as the average of the T>C respectively A>G exchange rate of reads mapping to the (+) respectivley (-) strand. The C>non-C probability ϵ was calculated as the average of the according, strand-specific base exchange rates.

From Equations (6) and (7), we have

$$\begin{aligned} \log P(o_j, h_j; \Theta) &= h_i \log \rho + (1-h_j)\log(1-\rho) + \log \binom{T_j}{o_j} \\ &\quad + o_j \log p_{h_j} + (T_j - o_j)\log(1-p_{h_j}) \end{aligned} \tag{2}$$

We employ an EM algorithm to estimate the ratio of newly synthesized RNAs ρ and the labeling efficientcy ℓ for each 3'UTR separately for each compartment and time point in each time series. To that end, we initiliased the labeliung efficiency l with p_0 and the $\frac{new}{total}$ RNA ratio $\rho$with the observed $\frac{labeled}{total}$ RNA ratio.

E-step. Let $H = (h_j; j = 1, ..., J)$, $H_{-1} = (h_j; j = 2, ..., J)$. Given some parameter set $\Theta' = (\rho'$,e'), we have to optimize the lower bound function $Q(\Theta; \Theta')$ with respect to $\Theta = (\rho, e)$. Using standard algebraic transformations of the lower bound function, we obtain

$$\begin{aligned} Q(\Theta; \Theta') &:= \mathbb{E}_{P(H|O;\Theta')} \log P(O, H; \Theta) \\ &= \sum_{H \in \{0,1\}^J} P(H \mid O; \Theta') \log P(O, H; \Theta) \\ &= \sum_{H_{-1}=(h_2,...,h_J) \in \{0,1\}^{J-1}} \sum_{h_1 \in \{0,1\}} (P(H_{-1} \mid O_{-1}; \Theta') \cdot P(o_1, h_1; \Theta')) \\ &\quad \cdot (\log P(H_{-1} \mid O_{-1}; \Theta) + \log P(o_1, h_1; \Theta)) \end{aligned} \tag{3}$$

$$
\begin{aligned}
&= \sum_{h_1 \in \{0,1\}} P(h_1 \mid o_1; \Theta') \log P(o_1, h_1; \Theta) \\
&\quad + \sum_{H_{-1} \in \{0,1\}^{J-1}} P(H_{-1} \mid O_{-1}; \Theta') \log P(O_{-1}, H_{-1}; \Theta) \\
&\overset{\text{induction}}{=} \sum_{j=1}^{J} \sum_{h_j \in \{0,1\}} P(h_j \mid o_j; \Theta') \log P(o_j, h_j; \Theta)
\end{aligned}
$$

Let

$$
c_{j,h_j} := P(h_j \mid o_j; \Theta') = \frac{P(o_j \mid h_j; \Theta') P(h_j; \Theta')}{\sum_{h_j \in \{0,1\}} P(o_j \mid h_j; \Theta') P(h_j; \Theta')} \quad , \ j = 1, ..., J \tag{4}
$$

and $C_0 = \sum_j c_{j,0}$ and $C_1 = \sum_j c_{j,1}$, $A = \sum_j c_{j,1} o_j$, $B = \sum_j c_{j,1}(T_j - o_j)$. Then, (3) simplifies to

$$
\begin{aligned}
Q(\Theta; \Theta') &= \sum_j c_{j,0} \left[\log(1-\rho) + \log \binom{T_j}{o_j} + o_j \log p_0 + (T_j - o_j) \log(1 - p_0) \right] \\
&\quad + \sum_j c_{j,1} \left[\log \rho + \log \binom{T_j}{o_j} + o_j \log p_1 + (T_j - o_j) \log(1 - p_1) \right] \\
&= C_0 \log(1-\rho) + C_1 \log \rho + A \log p_1 + B \log(1 - p_1) + \text{const}
\end{aligned} \tag{5}
$$

where all terms that do not depend on ℓ or ρ are summarized in the constant.

M-step. Taking the partial derivative of Q with respect to ρ and equating this expression to zero yields

$$
\begin{aligned}
0 = \frac{\partial Q(\Theta; \Theta')}{\partial \rho} &= -\frac{C_0}{1-\rho} + \frac{C_1}{\rho} \\
\rho &= \frac{C_1}{C_0 + C_1} = \frac{C_1}{J}
\end{aligned} \tag{6}
$$

Equating the partial derivative of Q with respect to ℓ to zero yields

$$
\begin{aligned}
0 = \frac{\partial Q(\Theta; \Theta')}{\partial \ell} &= \frac{A}{p_1} \cdot \frac{\partial p_1}{\partial \ell} + \frac{B}{1 - p_1} \cdot \frac{\partial (1 - p_1)}{\partial \ell} \\
&= \frac{A}{p_1} \cdot (1 - \epsilon - p_0) - \frac{B}{1 - p_1} \cdot (1 - \epsilon - p_0)
\end{aligned} \tag{7}
$$

Solving this for p_1 (assuming $1 - \epsilon - p_0 \neq 0$) and then solving Equation (1) for ℓ yields

$$
\begin{aligned}
p_1 &= \frac{B}{A + B} \\
\ell &= \frac{p_1 - p_0}{1 - \epsilon - p_0}
\end{aligned} \tag{8}
$$

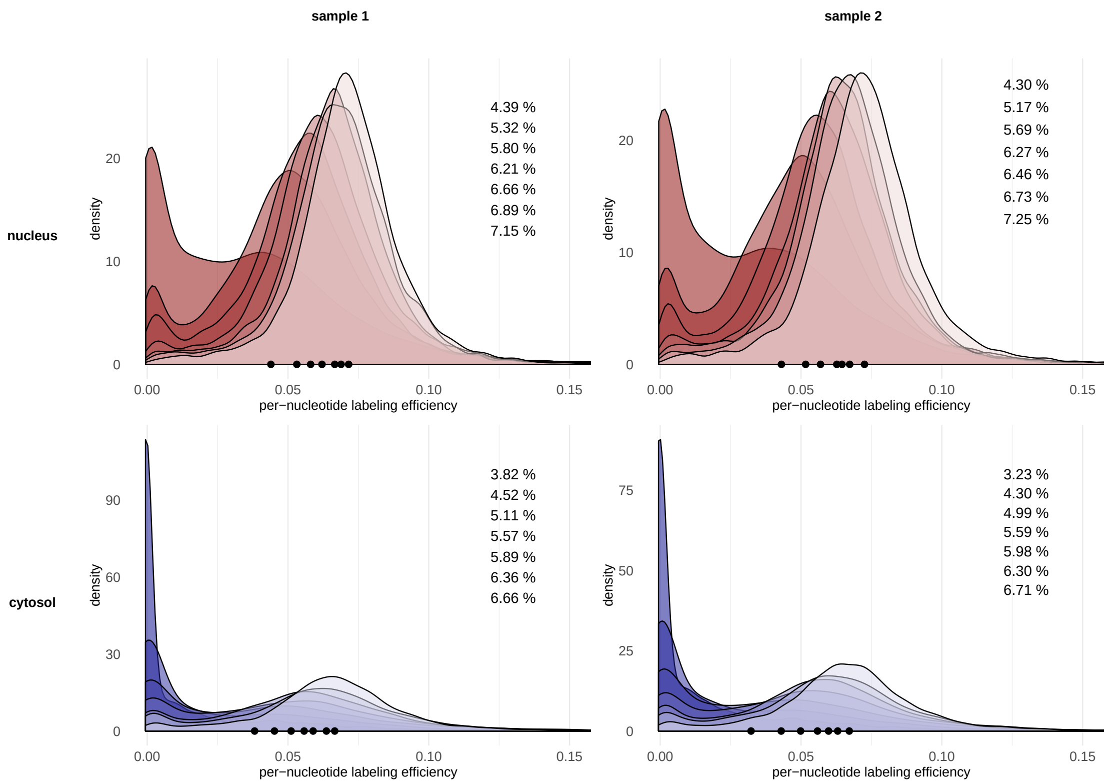

Fig H. **Distribution of labeling efficiency estimates ℓ, calculated for each 3'UTR and each compartment, time point and time series.** Each distribution represents one time point for the respective time series and compartment, with darker shades of a color encoding earlier and brighter shades encoding later time points. Efficiencies were estimated for all 3'UTRs with a coverage of at least 100 in the respective measurement. The black dots and text labels indicate the median labeling efficiencies obtained after discarding all 3'UTRs with an efficiency estimate smaller than 0.01 within a measurement. Text labels are ordered by increasing measurement time points. The x-axis is trimmed for better display.

6 Modeling of the labeling efficiency in the nucleus and the detection gap

General considerations on the labeling efficiency. The parameters of our model, such as RNA synthesis or RNA degradation, were so far assumed to be constant. While we have chosen minimally invasive procedures to keep these parameters unchanged, the assumption of constant labeling efficiency can be problematic, as discussed below. To understand this, remember that the time variable t, which appears in our dynamic equations, denotes the time after the start of 4sU labeling. When we say that a transcript has been

synthesized at time t, we mean that this transcript has been polyadenylated and can be detected with our experimental method at time t. Let $l(t)$ be the labeling efficiency at time t, i.e., the probability that a 4sU nucleotide instead of uridine is included in a nascent transcript at time t. Because it may take a while for the 4sU molecules to diffuse into the nucleus, we expect the labeling efficiency to increase monotonically from $l(0) = 0$ to a certain limit.

First, we verify the assumption implicit in our model that labeling efficiency is the same for each uridine position of the same transcript. The length of a primary human gene transcript (including introns) ranges between 0.5kb and $1,000$kb [6]. As the elongation speed of RNA Polymerase II ranges between 0.5kb/min and 5kb/min[7], the synthesis of a primary transcript can take up to a few hours. The onset of transcription may therefore even precede the start of 4sU labeling in long genes, which seems inconsistent with the assumption of constant labeling efficiency. However, this apparent contradiction resolves if we considered that we are sequencing only the 3' ends of mRNAs, i.e., the last ˜80nt upstream of the poly(A) tail of a transcript. All considerations on labeling efficiency refer only to this 3' end, which is synthesized by RNA polymerase II within seconds. It therefore seems justified to assume that the labeling efficiency during 3'-end synthesis of a transcript is constant and equals $l(t)$. Note that we still have neglected the time it takes until the poly(A) tail has been added after transcription termination. As a consequence, we will observe all processes described above with a delay. We call this delay the detection gap Δ, which we expect to be in the range of seconds to minutes.

Estimation of the labeling efficiency kinetics. Denote by $\text{RNA}_x^{new}(t)$ the amount of (mature) RNA of transcript x in the nucleus that has been newly synthesized (i.e., is detecteble by RNA-seq) within t minutes after the start of 4sU labeling. Let $\text{RNA}_x^{lab}(t)$ the amount of newly synthesized RNA that can be detected as labeled (by at least one T>C transition) within t minutes after labeling start. Our next goal is to estimate the fraction $f_x(t) = \text{RNA}_x^{lab}(t)/\text{RNA}_x^{new}(t)$ of newly synthesized RNA of a transcript x at time t which is recognized as labeled. This fraction will be used later as a correction factor in the estimation process. We assume that the labeling efficiency in the nucleus follows a delayed saturation kinetics,

$$l(t) = l(t) = \ell_{max} \cdot max(1 - e^{-\gamma(t-\Delta)}, 0) \tag{9}$$

Here, ℓ_{max} is the limit 4sU incorporation rate, γ models the diffusion speed of $4sU$ inside the cell, and Δ is the detection gap. Let $u_x > 1$ be the number of Uridines in the sequenced part of transcript x. The lengths of the sequenced reads are very similar, so without loss we may assume this number to be constant for each

transcript of type x. Then, the probability of a transcript x that has been newly synthesized exactly at time t to be recognized as labeled is

$$r_x(t) = 1 - (1 - l(t))^{u_x} \tag{10}$$

Assuming a constant synthesis rate μ_x and a constant export+decay rate λ_x for x, the ordinary differential equations for the nuclear RNA become

$$\begin{aligned} \frac{d}{dt}\mathrm{RNA}_x^{new}(t) &= \mu_x - \lambda_x \mathrm{RNA}_x^{new}(t) \\ \frac{d}{dt}\mathrm{RNA}_x^{lab}(t) &= \mu_x \cdot r_x(t) - \lambda_x \mathrm{RNA}^{lab}(t) \end{aligned} \tag{11}$$

They have the solutions

$$\begin{aligned} \mathrm{RNA}_x^{new}(t) &= \mu_x \frac{1 - e^{-\lambda_x t}}{\lambda_x} \\ \mathrm{RNA}_x^{lab}(t) &= \mu_x \int_0^t r_x(t') e^{\lambda_x (t'-t)} dt' \end{aligned} \tag{12}$$

Thus, the fraction $f_x(t)$ of newly synthesized RNAs that are detected as labeled/converted is

$$f_x(t) = \frac{\mathrm{RNA}_x^{lab}(t)}{\mathrm{RNA}_x^{new}(t)} = \frac{\lambda_x \cdot \int_0^t r_x(t') e^{\lambda_x (t'-t)} dt'}{1 - e^{-\lambda_x t}} \tag{13}$$

It remains to estimate s_{max} and γ. If t' is the time after labeling start at which a transcript x has been synthesized, the distribution of t' in the population of newly synthesized transcripts x at time t after labeling is

$$A_x(t'; t, \lambda_x) = e^{\lambda_x (t'-t)} / \int_0^t e^{\lambda_x (s-t)} ds = \lambda_x e^{\lambda_x t'} / (e^{\lambda_x t} - 1) \quad , \ t' \in (0, t] \tag{14}$$

We conclude that the average labeling efficiency measured for a single uridine in a newly synthesized transcript x at measurement time t is

$$e_x(t; \lambda_x, \gamma, \ell_{max}, \Delta) = \int_0^t l(t') A(t', t, \lambda_x) dt' \tag{15}$$

We have assessed how $e_x(t; \lambda_x, \gamma, \ell_{max})$ varies with transcript half lives, respectively their degradation rate λ_x (Fig Fig J). We found that for fixed time points t, e_x is approximately independent of λ_x, for λ_x corresponding to transcript half lives above $\sim$ 100min (i.e., relative changes are small). The reason is that for sufficiently

small λ_x, $A(t', t, \lambda_x)$ is approximately constant for all $t' \in (0, t]$ except for very small values of t'. We therefore define the (degradation-rate independent) labeling efficiency

$$\bar{e}(t; \gamma, \ell_{max}, \Delta) := \int_0^t l(t')dt' = \ell_{max}\left(1 - \frac{1 - e^{-\gamma(t-\Delta)}}{\gamma(t-\Delta)}\right) \tag{16}$$

and keep in mind that

$$\bar{e}(t; \gamma, \ell_{max}, \Delta) \approx e_x(t; \lambda_x, \gamma, \ell_{max}, \Delta) \tag{17}$$

for λ_x small enough, say for a transcript half life greater than 100min.

In a first round of our estimation procedure, we assume that labeling efficiencies do not depend on the degradation rate of a transcript. Using our estimation procedure (S1 Appendix Section 5), we obtain preliminary labeling efficiency estimates $\hat{e}_x(t)$ for each measurement time t and transcript x, together with preliminary half life estimates for each transcript x. We found that 98% of all transcripts have half life estimates above 100min. Since the median is a robust measure of location for skewed distributions, we use the median of all $\hat{e}_x(t)$ for transcripts with a preliminary half life estimate >100min as an estimate of $\bar{e}(t; \gamma, \ell_{max})$:

$$\hat{e}(t) := \text{median}\left(\hat{e}_x(t); \text{ prelim. half life}\lambda_x > 100\text{min}\right) \tag{18}$$

We additionally account for the detection gap by introducing a time shift of Δ for our observations. The estimates of γ, s_{max} and Δ are then obtained by a least squares fit of

$$\hat{e}(t) \sim \bar{e}(t; \gamma, \ell_{max}, \Delta) \quad , \; t \in \{15, 30, 45, 60, 90, 120, 180\text{min}\} \tag{19}$$

This yields (see Fig I)

$$\ell_{max} \;=\; 7.8\% \; (7.9\%) \tag{20}$$

$$\gamma \;=\; 0.069\text{min}^{-1} \; (0.056\text{min}^{-1}) \tag{21}$$

$$\Delta \;=\; -14\text{min} \; (-18\text{min}) \tag{22}$$

for replicate time series 1 (replicate time series 2). Contrary to our intuition, the estimated value for the detection gap was negative. We still keep our model, since it represents an excellent fit of labeling efficiency time curves.

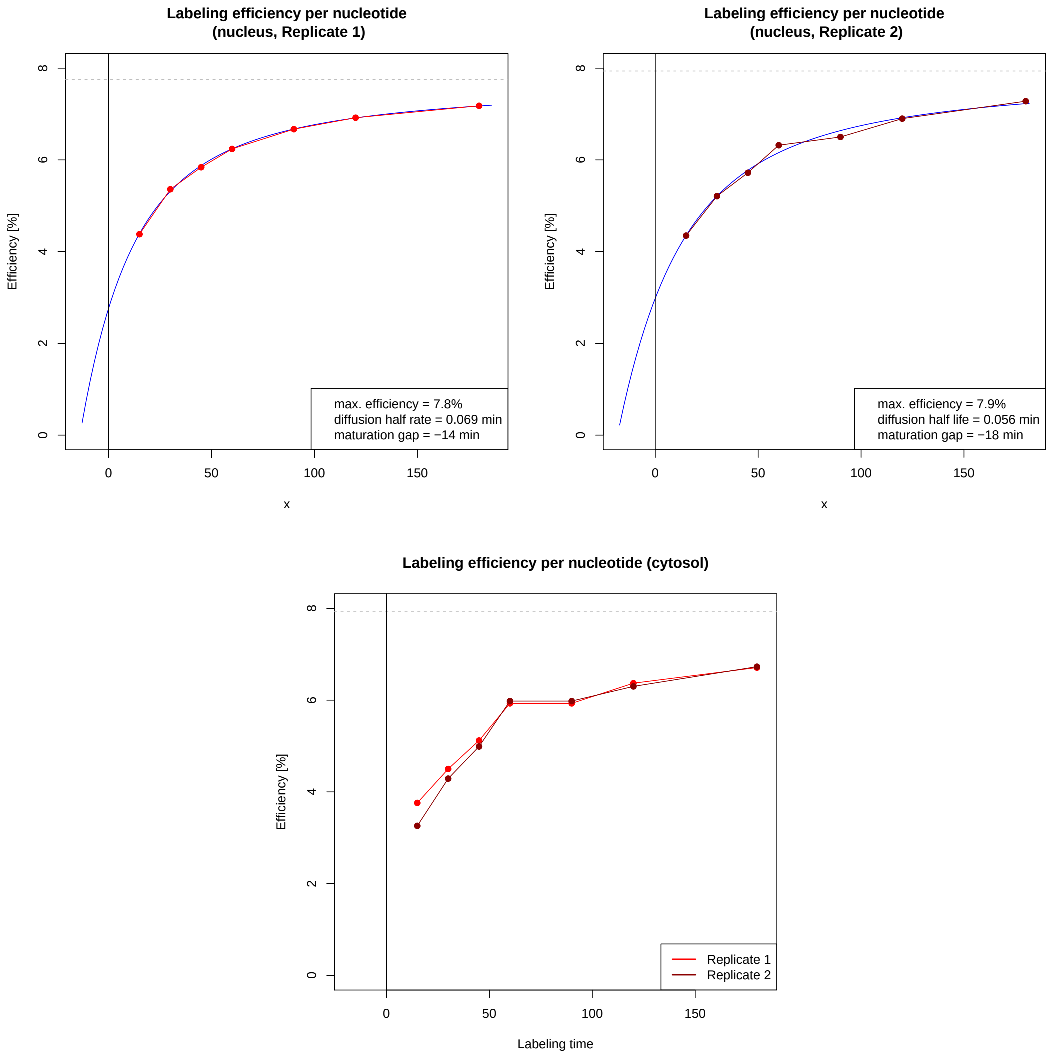

Fig I. **Per nucleotide labeling efficiencies.** The dots (red/dark red: replicate 1/replicate 2) show the median values for $\hat{e}(t)$ for each measurement time point (top: nucleus, bottom: cytosol). The blue lines are the least squares fits for the parameters γ, ℓ_{max} and Δ in both replicate time series. The parameters of the labeling efficiency function $\bar{e}(t - \Delta; \gamma, \ell_{max})$ (Equation (16)) were fitted according to Equation (19).

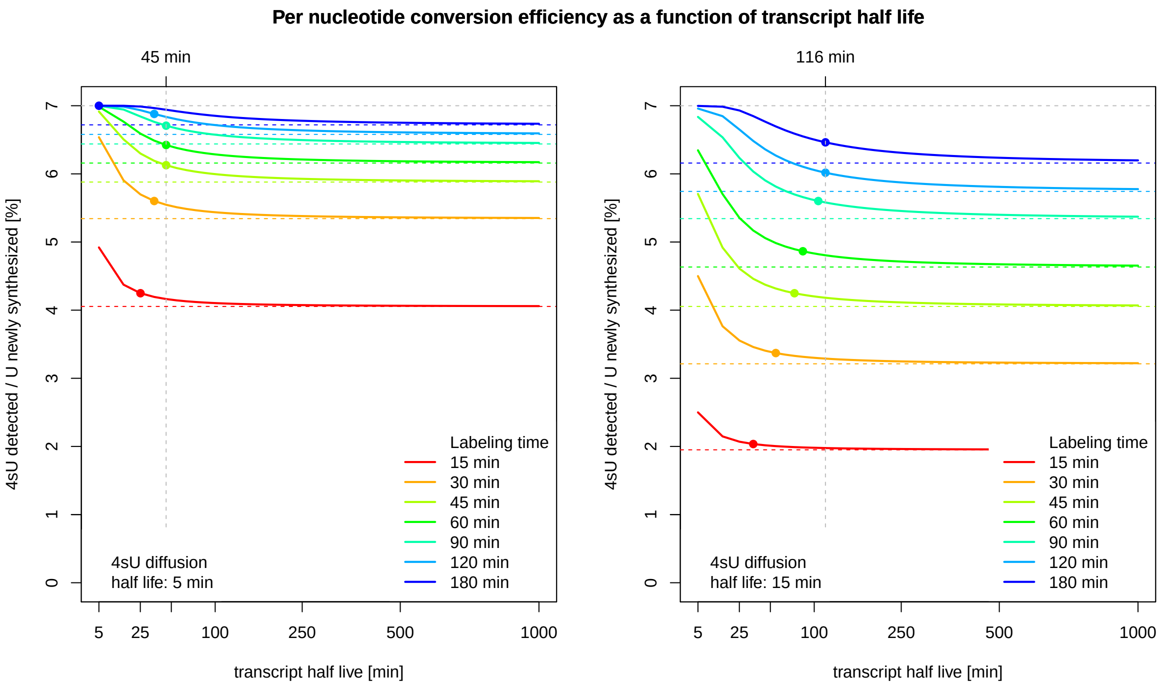

Fig J. **Per nucleotide labeling+conversion efficiency ($e(t;\lambda_x,\gamma,\ell_{max},g)$, see Equation (15)) as a function of transcript halflife ($\log(2)/\lambda_x$, x-axis).** This was done for a diffusion rate γ coresponding to a half live $(\log 2)/\gamma$ of 5min (left plot) and a half life of 15min (right plot), which is considered a realistic upper bound for γ. The maximum labeling efficiency was set to $\ell_{max} = 7\%$ (dashed gray horizontal lines). Each colored curve corresoponds to a certain labeling time $t = 15, 30, ..., 180$min (see the inset). The dashed colored horizontal lines indicate the corresponding values of $\bar{e}(t;\gamma,\ell_{max})$. The colored dots on the curves indicate the half lives above which Approximation (17) has an error smaller than 5%. The dashed gray vertial line at 45min (left) respectively 116min (right) indicates the transcript half life above which Approximation (17) has an error smaller than 5% for all measurement time points.

Necessity of modeling time-dependency of the labeling efficiency. So far, half life estimation procedures based on SLAM-Seq assumed a labeling efficiency that is constant over time [8]. This means replacing the above labeling efficiency estimate $f_x(t)$ by an approximation $g_x(t)$ assuming constant per-uridine labeling efficiency $\bar{e}_x(t)$:

$$g_x(t) = 1 - (1 - \bar{e}_x(t))^{u_x}$$

We have assessed whether this assumption causes a relevant bias, or whether this simplification is justified. Starting with the equation

$$\frac{\mathrm{RNA}_x^{lab}(t)}{\mathrm{RNA}_x^{total}(t)} = \frac{\mathrm{RNA}_x^{lab}(t)}{\mathrm{RNA}_x^{new}(t)} \cdot \frac{\mathrm{RNA}_x^{new}(t)}{\mathrm{RNA}_x^{total}(t)} = f_x(t) \cdot (1 - e^{-\lambda_x t})$$

we replace $f_x(t)$ by $g_x(t)$ in the estimation process. This amounts to letting

$$g_x(t) \cdot (1 - e^{-\hat{\lambda}_x t}) \approx \frac{\text{RNA}_x^{lab}(t)}{\text{RNA}_x^{total}(t)} = f_x(t) \cdot (1 - e^{-\lambda_x t})$$

If we merely use the observation at measurement time point t, the biased estimate $\hat{\lambda}_{x,t}$ of λ_x becomes

$$\hat{\lambda}_{x,t} = -\frac{1}{t} \log \left[1 - \frac{f_x(t)}{g_x(t)} \cdot \left(1 - e^{-\lambda_x t}\right) \right]$$

In a post-hoc analysis, we fix the values $\hat{\gamma}$ and $\hat{s}_{max}$ to the values obtained from replicate time series 1, see Equations (20,20). We then calculated the the relative bias $\hat{\lambda}_{x,t}/\lambda_x$ for all our measurement time points t, for the whole range of transcript half lives λ_x, and for various uridine contents of the reads (Fig K). The relative bias is generally negative for short half lives (except for very high uridine contents and very short labling times). The longer the labeling times, the more the bias is shifted towards negative values. Above a half life of ˜100min, the bias is asymptotically constant as a function of half life, and it ranges in the interval $[-7\%, 7\%]$, with longer labeling times resulting in smaller biases. In conclusion, the estimation bias relatively small for the setting in our experiment. We therefore do correct for the time-dependency of the labeling efficiency, but we generally advise to perform an assessment of this bias in future SLAM-Seq experiments.

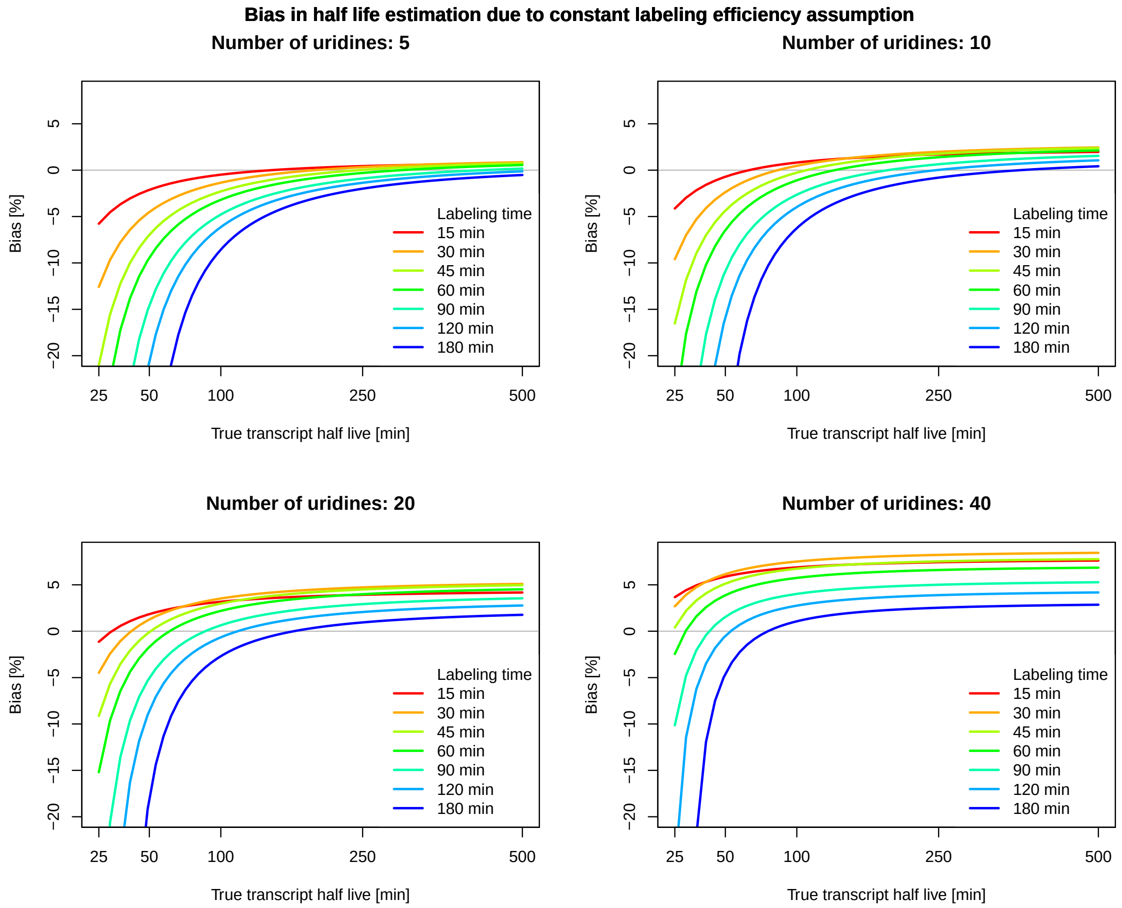

Fig K. **Assessment of half life estimation bias when assuming constant labeling efficiency.** Each line shows the relative bias (in %) as a function of the (true) transcript half life. Line colors correspond to different labeling times (see insets). The caclulations were done for different uridine contents of a transcript: 5 (top left), 10 (top right), 20 (bottom left), 40 (bottom right) uridines per sequenced read. The time dependency of the labeling efficiency was modeled by Equation (9) with parameters $\gamma = 0.069\text{min}^{-1}$ and $\ell_{max} = 7.8\%$ estimated from replicate time series 1.

7 Effects of variance stabilization

We thoroughly assessed the effects of variance stabilization of the estimates $\frac{new}{total}$RNA ratios as shown in Fig L.

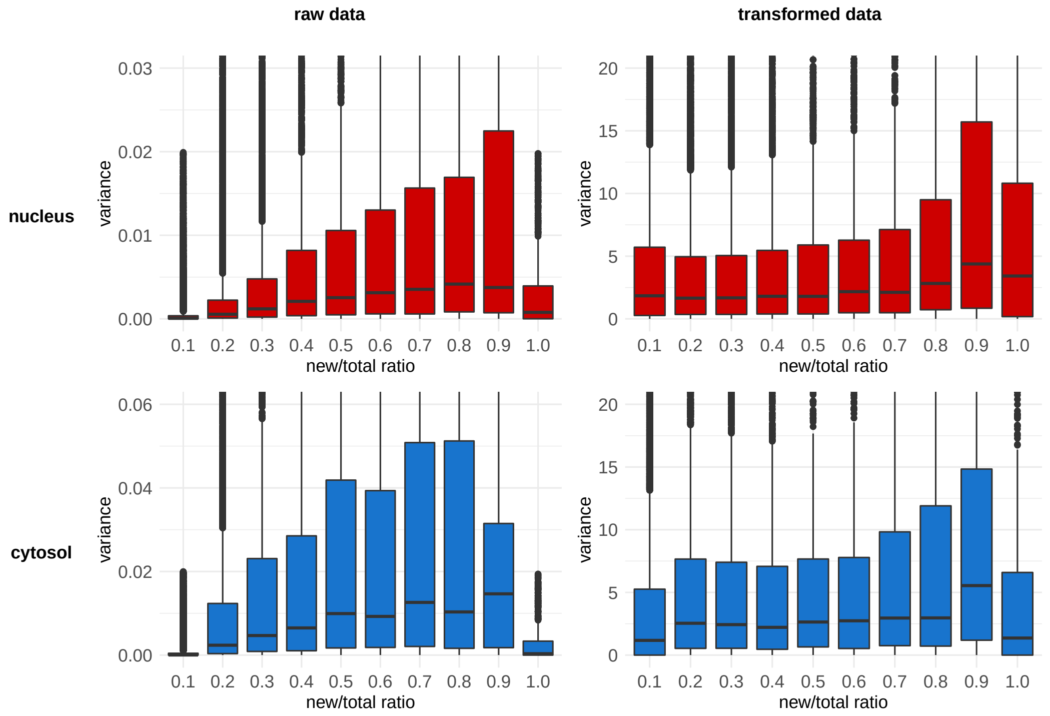

Fig L. **Impact of variance stabilizing transformation.** Left: Variance of new/total mRNA ratios (y-axis) as a function of the mean (x-axis, binned in steps of 0.1), for the nuclear (red) and cytosolic (blue) fraction. Variance was calculated as $\frac{1}{2}(\rho_1 - \rho_2)^2$, where ρ_i are the estimated new/total ratios from the two replicate time series, for all time points and all transcripts. Right: Same as left, with ρ_i replaced by $\sqrt{4N_i}\text{arcsin}\sqrt{\rho_i}$.

8 T>C conversion and mismatch statistics

Conversions originating from the labeling are expected to show as A>G on the (-) and T>C on the (+) strand. Expectedly and reassuringly, the labeling conversion rates clearly show an increasing trend over time, while the other rates stay constant and low. Also, the cytosolic rates increase less and slower than the nuclear rates, as the export of newly synthesized, labeled RNA transcripts from the nucleus to the cytosol requires time.

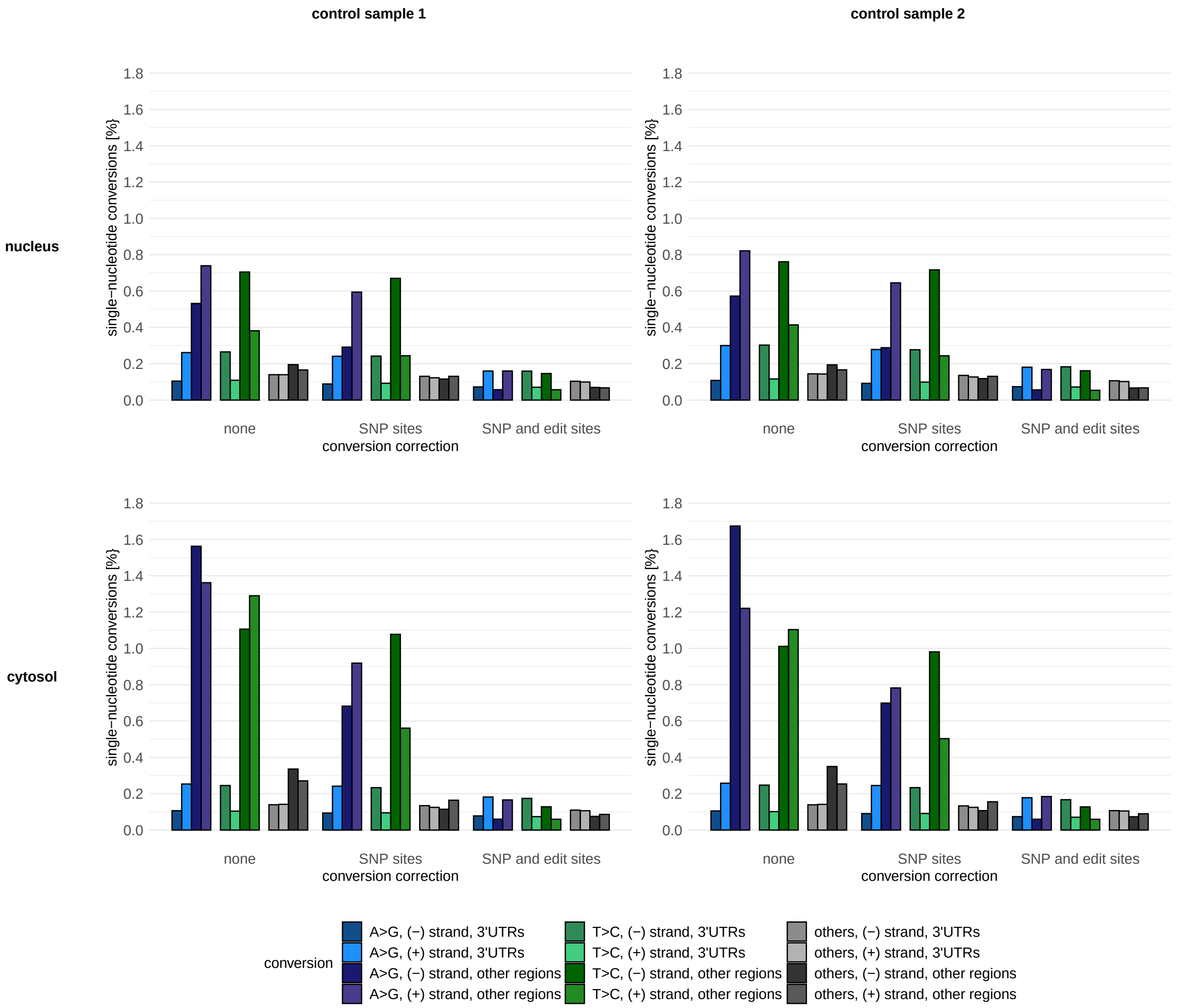

Fig M. **Conversion statistics of the control samples. No conversion correction: mismatch rates as observed in the primary alignment.** SNP-sites correction: mismatch rates as observed in the primary alignment when excluding all nucleotide positions annotated as known SNP site. SNP and edit-site correction: conversions as observed in the primary alignment when excluding all nucleotide positions annotated as known SNP site or tagged as potential editing sites. A>G (T>C) conversions: A>G (T>C) conversions as observed in the read alignment. Other conversions: average of all non-A>G and non-T>C conversions observed in the read alignment. Conversions of (-) strand ((+) strand) 3'UTRs: conversions of all uniquely mapped reads which are assigned to an annotated (-) strand ((+) strand) 3'UTR. Conversions of other regions on the (-) strand ((+) strand): conversions of all uniquely mapped reads which are assigned to a non-3'UTR peak on the (-) strand ((+) strand) with a minimum coverage of 5 in the sample and compartment.

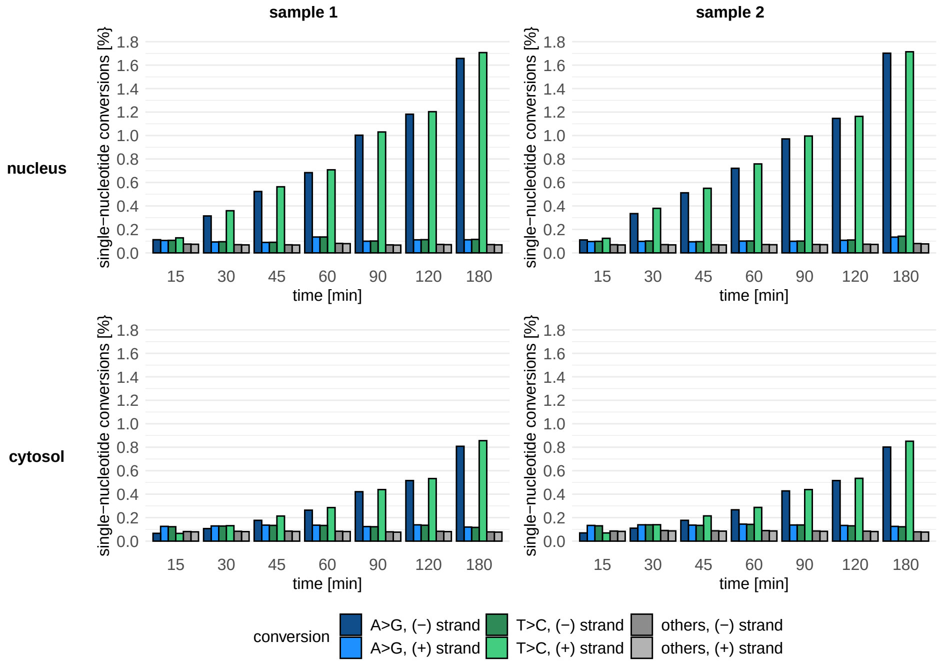

Fig N. **Conversion statistics of the SLAM-seq labeling samples, shown for the reads assigned to annotated 3'UTRs.** Statistics are corrected for both annotated SNP sites and potential editing sites. A>G (T>C) conversions: A>G (T>C) conversions as observed in the read alignment. Other conversions: average of all non-A>G and non-T>C conversions observed in the read alignment. Conversions of the EM algorithm(-) strand ((+) strand): conversions of all uniquely mapped reads which are assigned to an annotated (-) strand ((+) strand) 3'UTR.

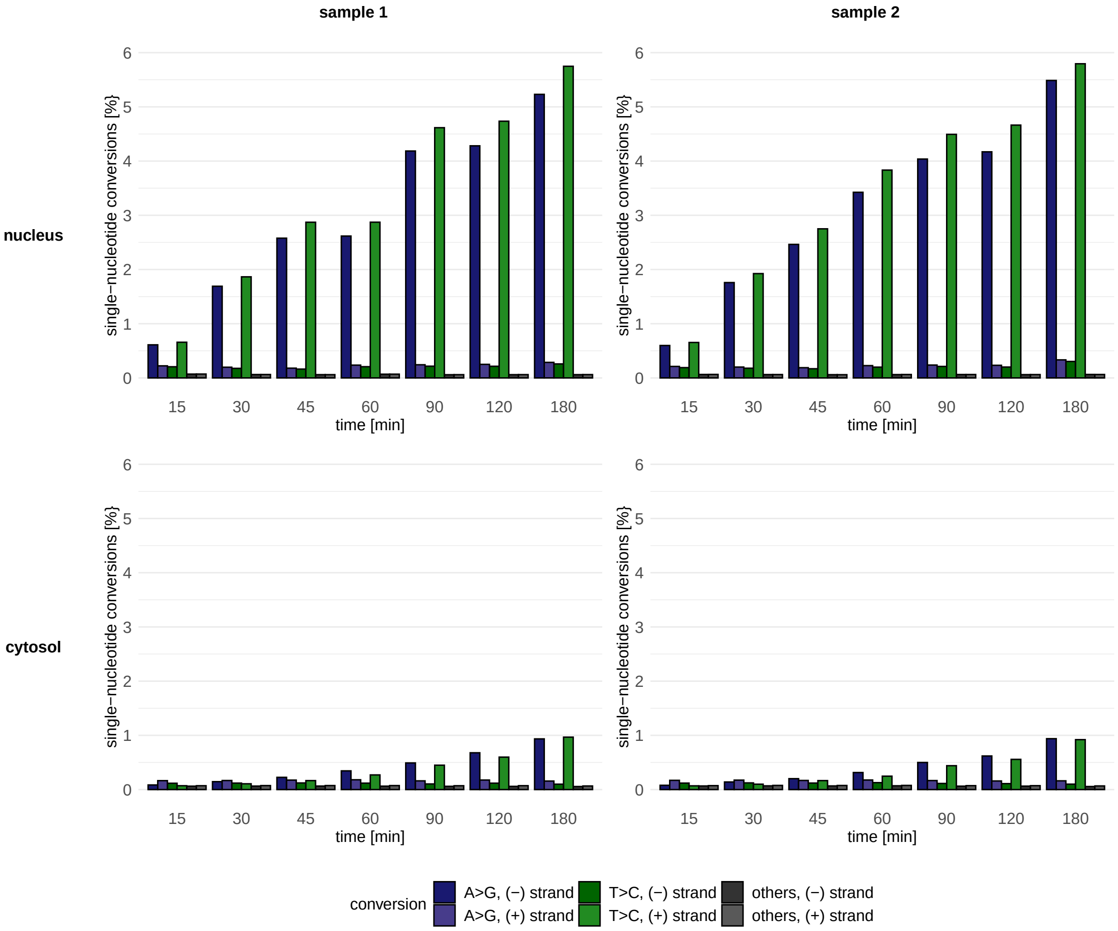

Fig O. **Conversion statistics of the SLAM-seq labeling samples, shown for the reads assigned to non-3'UTR peaks.** For each measurement, peaks with a coverage of less than 5 in that measurement were excluded from the calculations. Statistics are corrected for both annotated SNP sites and potential editing sites. A>G (T>C) conversions: A>G (T>C) conversions as observed in the read alignment. Other conversions: average of all non-A>G and non-T>C conversions observed in the read alignment. Conversions of the (-) strand ((+) strand): conversions of all uniquely mapped reads which are assigned to an annotated (-) strand ((+) strand) non-3'UTR peak.

References

[1] Neumann T, Herzog VA, Muhar M, von Haeseler A, Zuber J, Ameres SL, et al. Quantification of experimentally induced nucleotide conversions in high-throughput sequencing datasets. BMC Bioinformatics. 2019 May;20(1):258. Available from: https://doi.org/10.1186/s12859-019-2849-7.

[2] Langmead B, Salzberg SL. Fast gapped-read alignment with Bowtie 2. Nature Methods. 2012 Apr;9(4):357-9. Number: 4 Publisher: Nature Publishing Group. Available from: https://www.nature.com/articles/nmeth.1923.

[3] Kim D, Langmead B, Salzberg SL. HISAT: a fast spliced aligner with low memory requirements. Nature Methods. 2015 Apr;12(4):357-60. Number: 4 Publisher: Nature Publishing Group. Available from: https://www.nature.com/articles/nmeth.3317.

[4] Karolchik D, Hinrichs AS, Furey TS, Roskin KM, Sugnet CW, Haussler D, et al. The UCSC Table Browser data retrieval tool. Nucleic Acids Research. 2004 Jan;32(Database issue):D493-6.

[5] O'Leary NA, Wright MW, Brister JR, Ciufo S, Haddad D, McVeigh R, et al. Reference sequence (RefSeq) database at NCBI: current status, taxonomic expansion, and functional annotation. Nucleic acids research. 2016;44(D1):D733-45.

[6] Grishkevich V, Yanai I. Gene length and expression level shape genomic novelties. Genome Research. 2014 Jan;24(9):1497-503. Company: Cold Spring Harbor Laboratory Press Distributor: Cold Spring Harbor Laboratory Press Institution: Cold Spring Harbor Laboratory Press Label: Cold Spring Harbor Laboratory Press Publisher: Cold Spring Harbor Lab. Available from: https://genome.cshlp.org/content/24/9/1497.

[7] Jonkers I, Kwak H, Lis JT. Genome-wide dynamics of Pol II elongation and its interplay with promoter proximal pausing, chromatin, and exons. eLife. 2014 Apr;3:e02407. Publisher: eLife Sciences Publications, Ltd. Available from: https://doi.org/10.7554/eLife.02407.

[8] Herzog VA, Reichholf B, Neumann T, Rescheneder P, Bhat P, Burkard TR, et al. Thiol-linked alkylation of RNA to assess expression dynamics. Nature Methods. 2017 Dec;14(12):1198-204. Number: 12 Publisher: Nature Publishing Group. Available from: https://www.nature.com/articles/nmeth.4435.
