# Supplementary material for: Nuclear export is a limiting factor in eukaryotic mRNA metabolism
Source: PLoS Comput Biol. 2024 May 16;20(5):e1012059. doi: 10.1371/journal.pcbi.1012059 (PMC11135743; doi:10.1371/journal.pcbi.1012059)
Supplement: S2 Appendix — (PDF) [file pcbi.1012059.s002.pdf]

S2 Appendix

1 Assessment of parameter estimate variability

To check the variability in our parameter estimates, we correlated the assigned read counts per 3'UTR between time points, as well as the estimated $\frac{new}{total}$ RNA ratios and RNA half-lives of both time series replicates. This was done seperately for the nuclear and cytosolic half-life estimates (Fig A-Fig D).

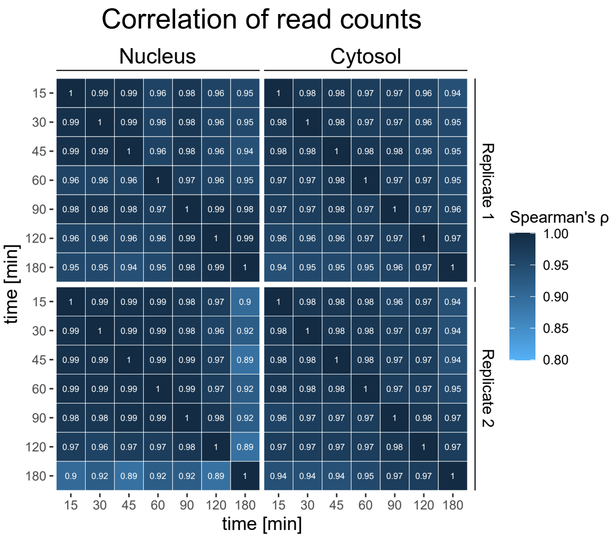

Fig A. **Correlation between new/total RNA ratio estimates obtained from two distinct SLAM-seq time-series.**

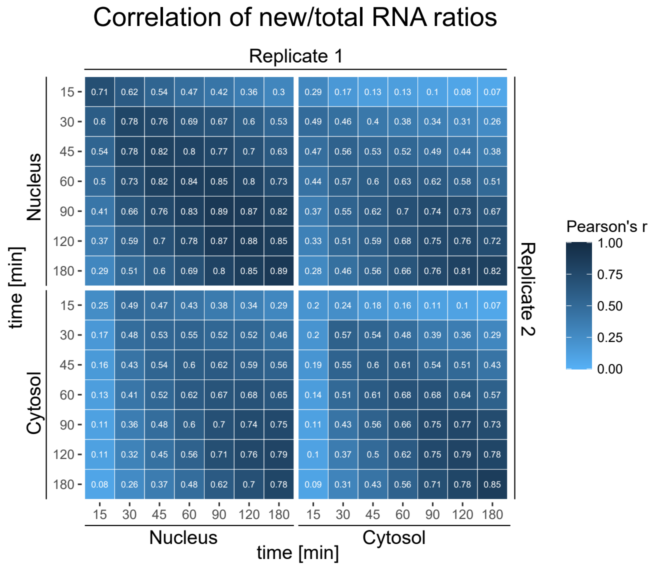

Fig B. **Correlation between new/total RNA ratio estimates obtained from two distinct SLAM-seq time-series.**

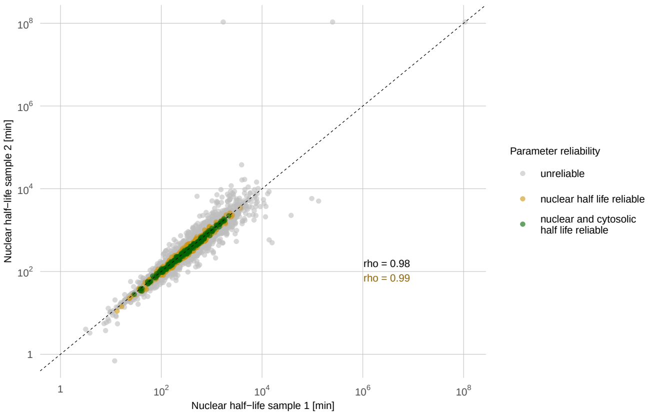

Fig C. **Correlation between nuclear half-lives obtained from two distinct SLAM-seq time-series.**

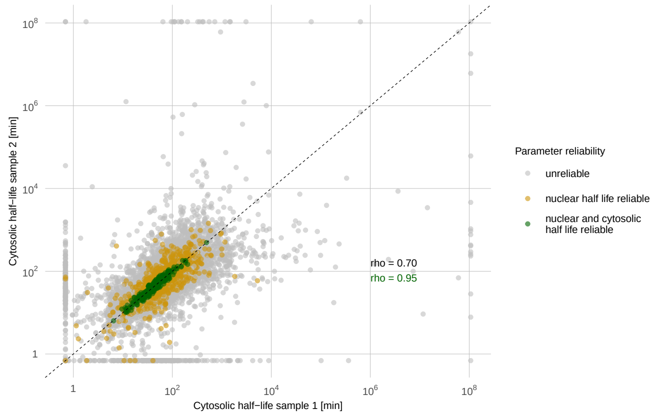

Fig D. **Correlation between cytosolic half-lives obtained from two distinct SLAM-seq time-series.**

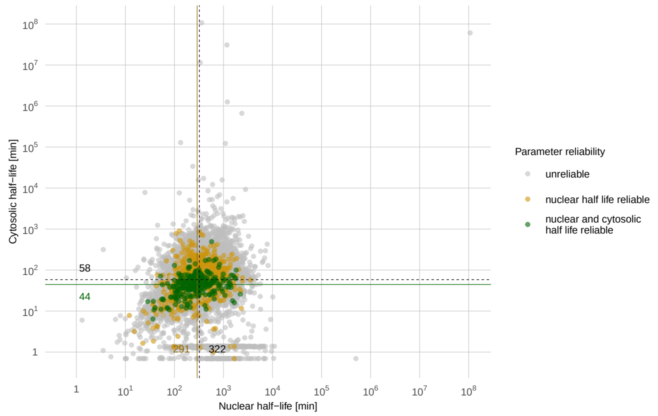

Fig E. **Nuclear and cytosolic RNA half-life estimates of 3'UTRs.** Half-lives were averaged over both measured time series. Stringent quality criteria were applied to score the reliability of the estimates. gray dots represent unreliable estimates, yellow dots correspond to estimates that passed additional reliability criteria in the nucleus, and green dots portray estimates that matched the reliability criteria in the nuclear and cytosolic compartment.

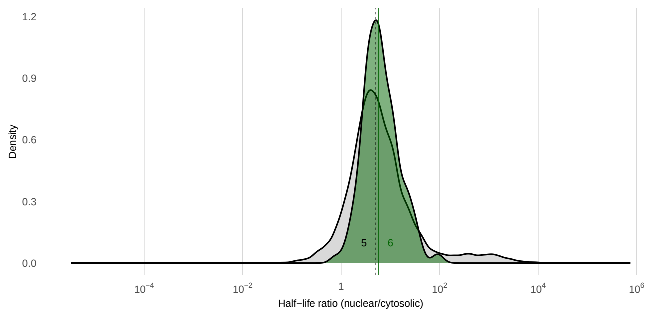

Fig F. **Nuclear by cytosolic half-life ratios of all 3'UTRs and 3'UTRs with reliable half-life estimates for both compartments.** The dashed and solid line indicate the median half-lives of all 3'UTRs and the 3'UTRs with reliable estimates, respectively.

2 Comparison with half-life estimates from literature

The compartment-specific parameter estimates for a certain transcript can be converted into an estimate of its RNA half life / degradation rate in the entire cell. Let $N_\infty = \frac{\mu}{\tau+\nu}$ and $C_\infty = N_\infty \cdot \frac{\tau}{\lambda}$ the RNA abundances of a certain transcript in the nucleus respectively cytosol. Assuming a one-compartment model with total steady-state RNA abundance $N_\infty + C_\infty$, a constant synthesis rate μ and degradation rate λ_w, the steady-state relation is

$$\frac{\mu}{\lambda_w} = N_\infty + C_\infty = \mu \left(\frac{1}{\tau+\nu} + \frac{1}{\lambda} \cdot \frac{\tau}{\tau+\nu} \right)$$

Assuming $\nu \approx 0$, the whole-cell half life h_w is simply the sum of the nuclear half life h_n and the cytosolic half life h_c:

$$h_w := \frac{\log 2}{\lambda_w} = \frac{\log 2}{\nu+\tau} + \frac{\log 2}{\lambda} \cdot \frac{\tau}{\nu+\tau} \approx h_n + h_c$$

This surrogate half life h_w is used to compare our results with half life estimates from the literature.

For comparison of our RNA half-lives with estimates from the literature, we summarized our nuclear and cytosolic half-life estimates. These pseudo-WCE half-lives where correlated against estimates from Schueler et al. (2014) [1] (Fig 2C, Fig G). To that end, we averaged their replicates for both the MCF7 and HEK293 cell lines.

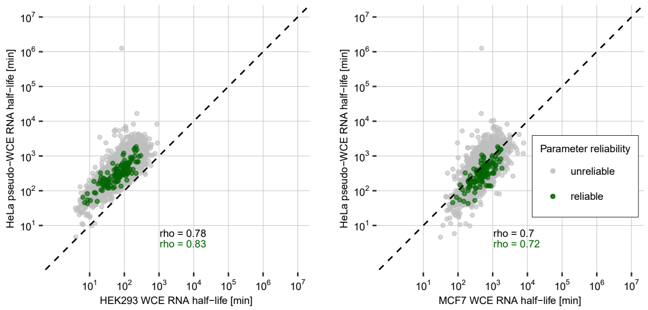

Fig G. **RNA half-life comparison between estimates obtained by our two-compartment model and estimates from the literature [1].** For comparison, pseudo-WCE half-lives were calculated by taking the sum of our nuclear and cytosolic half-life estimates.

Further, we also correlated our pseudo-WCE estimates against WCE RNA half-lives from Wu et al. (2019) [2] (Fig H) and cytosolic RNA half-lives from Zuckerman et al. (2020) [3] (Fig I).

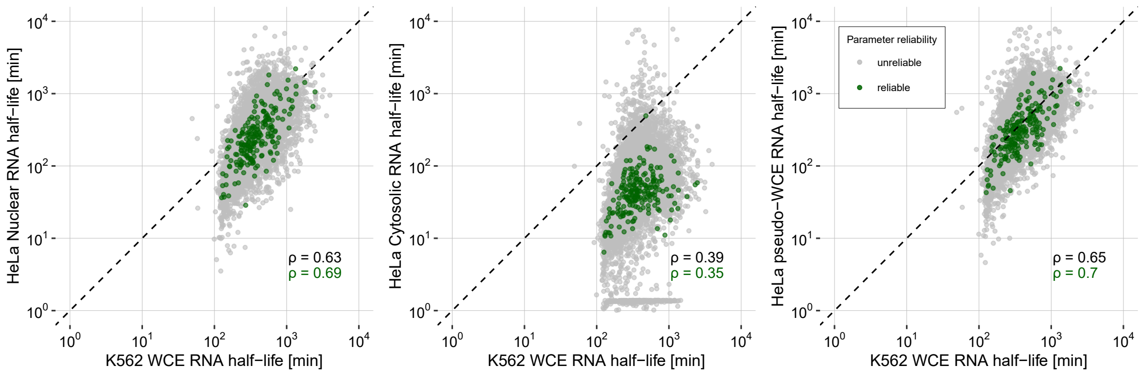

Fig H. **Comparison between RNA half-lives from a two-copartment model and literature estimates obtained by a one-compartment model fit to SLAM-seq whole-cell extracts [2].** Comparison between RNA half-life estimates by our two-compartment model and whole-cell extract half-life measurements from Wu et al. (2019) [2]. The nuclear and cytosolic RNA estimates were summed to generate pseudo-whole-cell estimates. Gray dots represent unreliable estimates, green points represent reliable estimates.

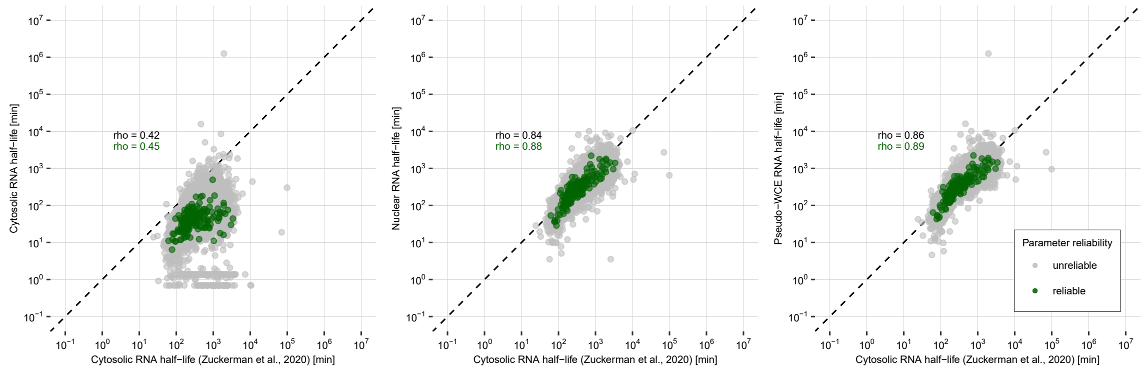

Fig I. **Comparison between RNA half-lives from a two-copartment model and estimates obtained by a one-compartment model fit to cytosolic fraction only [3].**

3 Cytosolic / nuclear RNA ratio estimation

For a set G of transcripts/3'UTRs/peaks considered, let t_g,n_g, c_g the relative abundances of the total, nuclear and cytosolic RNA population of $g \in G$, respectively (where the abundance is relative to all total/nuclear/cytosolic transcripts that belong to G, respectively, i.e., $\sum_{g \in G} t_g = \sum_{g \in G} n_g = \sum_{g \in G} c_g = 1$).

Spherical median regression. The set G here is all 3'UTRs with at least 50 counts in the total fraction of control sample (time $t = 0$min). There are 2662 such 3'UTRs, corresponding to a fraction of 4.3% of all annotated 3'UTRs. The selected 3'UTRs account for 84% of all reads assigned to 3'UTRs. If T,N and C are numbers that transform total/nuclear/cytosolic relative abundances to (average) molecule numbers per cell, the nuclear and the cytosolic fraction need to sum to the total fraction,

$$Tt_g = Nn_g + Cc_g \quad , \; g \in G$$

Scaling $(T, -N, -C)$ to length 1 and parameterizing them in angular coordinates, the resulting regression problem becomes

$$0 \sim (\sin\phi, \cos\phi \sin\psi, \cos\phi\cos\psi) * (t_g, n_g, c_g)^T$$

with an azimuth angle $\psi \in [0, 2\pi]$ and an altitude angle $\phi \in [0, \pi/2]$. This means that $v^T = (T, -N, -C)$ is orthogonal to the plane spanned by the expression vectors $x_g = (t_g, n_g, c_g)^T$, $g \in G$, i.e., $v^T x_g = 0$. The vector v is only determined up to scaling, but this is enough to uniquely determine the nuclear/cytosolic

ratio r of the total number of molecules,

$$r = \frac{\sum_g N n_g}{\sum_g C c_g} = \frac{N \sum_g n_g}{C \sum_g c_g} = \frac{N}{C}$$

We might therefore perform a linear regression to determine v, but this would lead to wrong results, as too many assumptions of linear regression are violated (to name just a few: the data is not drawn from a normal distribution, it is not homoschedastic, there are frequent outliers, linear regression assumes an error in the endpoint and not in the covariates while this is just the other way round here). Thus, we apply a robust, non-parametric regression procedure which resembles the Theil-Sen estimator in standard linear regression: We randomly pick three different transcripts from G and calculate the normal vector of the plane spanned by these three points in $\mathbb{R}^3$. Doing this $R = 10^5$times gives R normal directions represented by unit vectors v_i, $i = 1, ..., R$, $\|v_i\| = 1$. Our estimate is then obtained as the geometric median $\bar{v}$ of the v_i, where the median is defined as the (a) point minimizig the average distance of the v_i, $i = 1, ..., R$, to $\bar{v}$ according to some metric d,

$$\bar{v} = \underset{v \in \mathbb{R}^3, \|v\|=1}{\operatorname{argmin}} \frac{1}{R} \sum_{i=1}^{R} d(v_i, v) \tag{1}$$

In Euclidean space, $d(x, y) = \|x - y\|$, and finding $\bar{v}$ in Equation (1) is known as Fermat's problem, and the result $\bar{v}$ is called the geometric median. The resulting loss function is convex, and a fast algorithm to solve the minimization task is a gradient descent procedure known as Weiszfeld algorithm ([4], see [**?**] for a concise treatment). However, since the unit vectors merely define a direction, they are better represented as points in projective space $\mathbb{P}^2 = (\mathbb{R}^3 \setminus \{0\}) / \sim$, where $x \sim y$ if $x = \alpha y$ for some $\alpha \in \mathbb{R} \setminus \{0)$. Euclidean distance is not a meaningful distance measure in $\mathbb{P}^2$. Therefore, we use the Fubini-Study metric induced by the Euclidean scalar product in $\mathbb{R}^3$. This amounts to measuring the angular distance between (the smaller angle) between two elements x and y, $d(x, y) = \arccos \left| \frac{\langle x, y \rangle}{\|x\| \|y\|} \right|$. It can be shown that a similar optmization procedure leads to the desired results for Riemannian manifolds of curvature ≤ 2, and hence also for $\mathbb{P}^2$ with the Fubini-Study metric [5, 6]. In our case, the geometric median can easily be found using Nelder-Mead optimization (function optim in R). The result of the procedure is shown in Main Fig 2E. There, we chose an angular parametrization of the unit vectors to visualize them on a 2d plane: Let $v = (v_1, v_2, v_3) \in \mathbb{R}^3$, $\|v\| = 1$. Then there exists an azimuth angle $\psi \in [0, 2\pi)$ and an altitude angle $\phi \in [0, \pi/2]$ such that $\pm v = (\sin\phi, \cos\phi \cdot \sin\psi, \cos\phi \cdot \cos\psi)$. If $v_1 \neq 0$, this representation is unique. For $v_1 = 0$, we have $\phi = 0$, and if require $\psi \in [0, \pi)$, the choice of ψ is also unique in this case. This defines a map $v \mapsto (\psi, \phi) \in [0, 2\pi) \times [0, \pi/2]$ which serves for visualization. Vectors $v = (T, -N, -C)$ with the same $\frac{cyt}{nuc}$ RNA ratio map to $(\psi = \arctan \frac{N}{C}, \phi)$, and contour lines of

constant N/C ratio can be added to the plot for convenience.

Nuclear degradation rate as a function of the $\frac{cyt}{nuc}$ RNA ratio. Here, set G consists of the 251 3'UTRs for which cytosolic degradation rate λ_g and the nuclear removal rate $\tau_g + \nu_g$ could be estimated reliably (Material and Methods). In steady state, Equation (6) yields

$$\frac{Cc_g}{Nn_g} = \frac{\tau_g}{\lambda_g}$$

Multiplying by λ_g and adding ν_g on both sides leads to

$$\frac{C}{N} \cdot \frac{c_g}{n_g}\lambda_g + \nu_g = \tau_g + \nu_g$$

Substituting the nuclear/cytosolic RNA ratio $r = N/C$, we obtain an estimator of the nuclear degradation rate, $\hat{\nu}_g(r)$, as a function of r:

$$\hat{\nu}_g(r) = (\tau_g + \nu_g) - r^{-1}\frac{c_g}{n_g}\lambda_g \tag{2}$$

This allows us to calculate the tail probabilities of the empirical distribution of $\{\hat{\nu}_g(r); g \in G\}$,

$$P(\hat{\nu}(r) > 0) = \frac{|\{g \in G \mid \hat{\nu}_g(r) > 0\}|}{|G|}$$

as a function of r. As $\nu_g \geq 0$ for all g, we expect at least 50% of the estimates to be non-negative. According to Main Fig 2F, this is only the case for $r \leq 0.22$. For ratios $r \geq 1$, less than 6% of all nuclear degradation rate estimates would be positive.

Spike-in normalisation. Spike-ins containing constant, well-defined amounts of unique non-host RNAs were added to the nuclear and cytosolic fractions. Let G all 3'UTRs with at least 1 read count in each time point measurement of the time series, plus the set of all spike-in RNAs. For each RNA sample, we calculated a robust average $\bar{n}$ of the relative abundances for the nucleus, n_h, $h \in H$, as the interquartile mean, i.e. the mean of the central 50% of all relative expression values lying between the 25% and the 75% quantile of the data. Similarly, $\bar{c}$ was defined as the interquartile mean of c_h, $h \in H$. Let $\bar{n}_{\text{spike}}$ respectively $\bar{c}_{\text{spike}}$ the mean expression values of the spike-in RNAs we used in the nucleus respectively in the cytosol. As above, let N respectively C the constants that transfer relative abundances in the nucleus respectively the cytosol to molecules per cell. As the total amount of the spike-ins that has been added to each fraction is constant.

It can be calculated as $N\bar{n}_{\text{spike}} = C\bar{c}_{\text{spike}}$. An estimate of the $\frac{cyt}{nuc}$ RNA ratio is therefore obtained by

$$\hat{r} = \frac{C\bar{c}}{N\bar{n}} = \frac{\bar{c}}{\bar{n}} \cdot \frac{C\bar{c}_{\text{spike}}}{N\bar{n}_{\text{spike}}} \cdot \frac{\bar{n}_{\text{spike}}}{\bar{c}_{\text{spike}}} = \frac{\bar{c}}{\bar{n}} \cdot \frac{\bar{n}_{\text{spike}}}{\bar{c}_{\text{spike}}}$$

One such $\frac{cyt}{nuc}$ RNA ratio estimate $\hat{r}$ was calculated for each time point of each time series; the estimates ranged from 0.28 to 0.70.

4 Retrieval of gene-specific features

GENCODE annotation was used to retrieve information about exon counts and lengths, as well as CDS lengths. If a gene encodes for multiple transcript isoforms, these lengths and counts were averaged. We retrieved information about 3'UTR lengths from our UTR annotation file. Information about a transcript's GC content and length was retrieved from Ensembl biomart. We always considered the longest transcript isoform of a gene for comparison, assuming this transcript to be the dominantly expressed one. For each 3'UTR we measured in our experiments, we associated the corresponding half-life estimates with the gene-specific features via their gene names. To avoid ambiguity, 3'UTRs with multiple gene annotations were removed from the analyses.

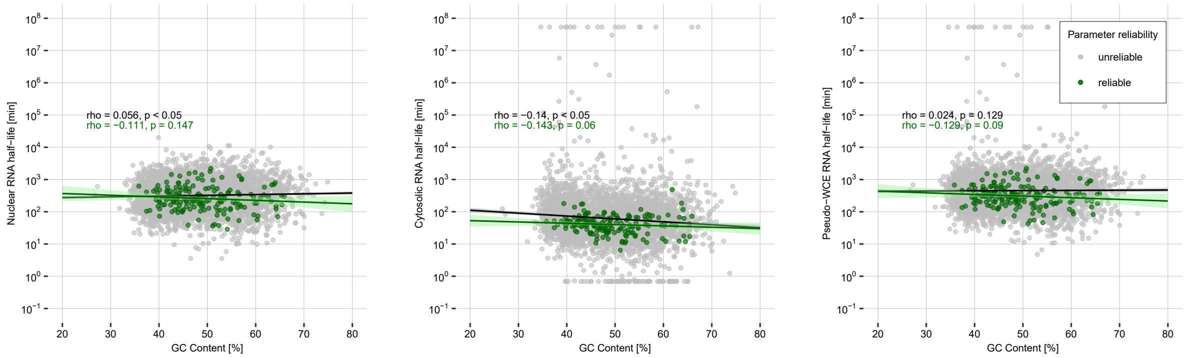

Fig J. **Scatterplot comparing the half-life estimates of our two-compartment model with the corresponding GC content of a respective gene.**

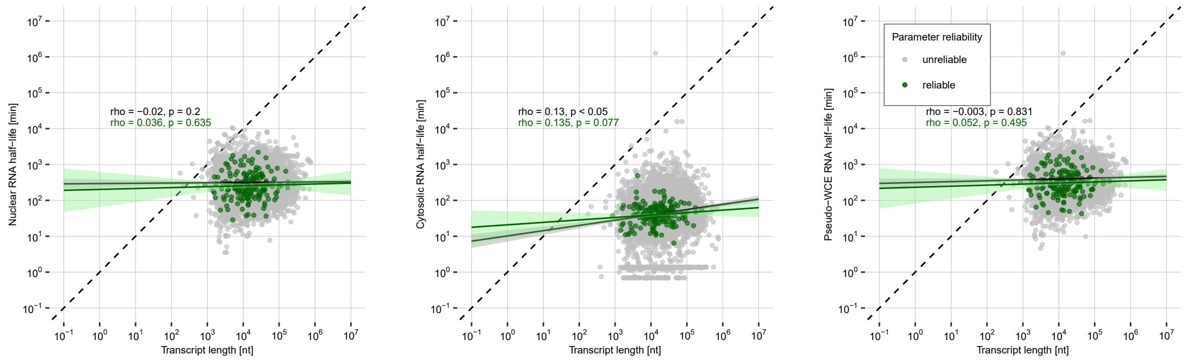

Fig K. **Scatterplot comparing the half-life estimates of our two-compartment model with the corresponding transcript length of a respective gene.**

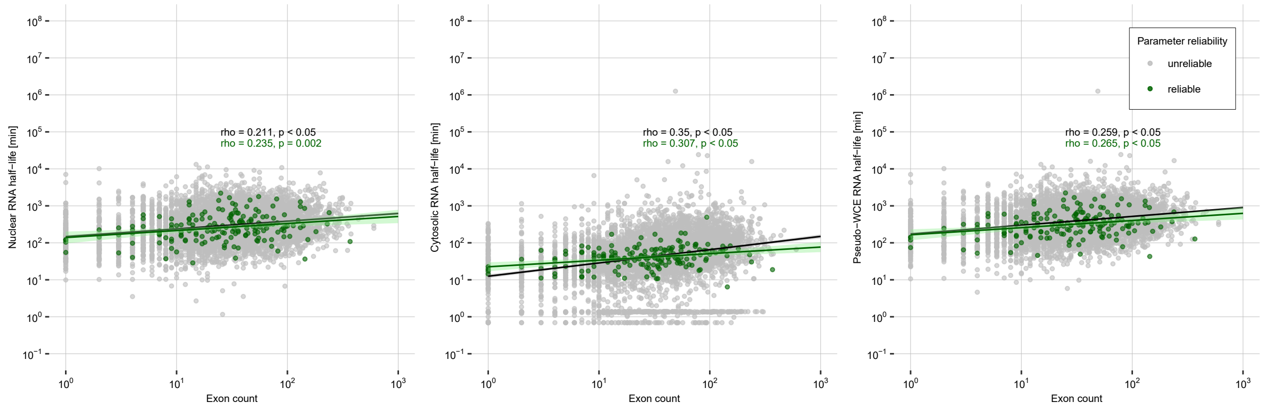

Fig L. **Scatterplot comparing the half-life estimates of our two-compartment model with the corresponding exon count of a respective gene.**

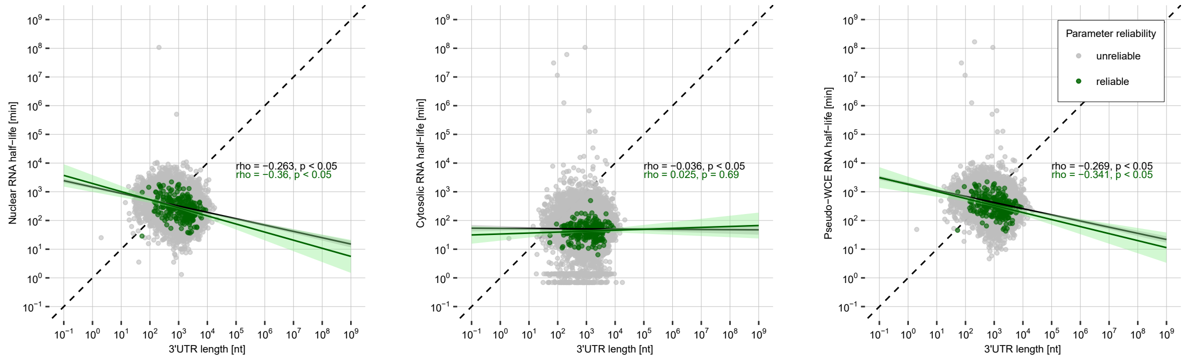

Fig M. **Scatterplot comparing the half-life estimates of our two-compartment model with the corresponding 3'UTR length of a respective gene.**

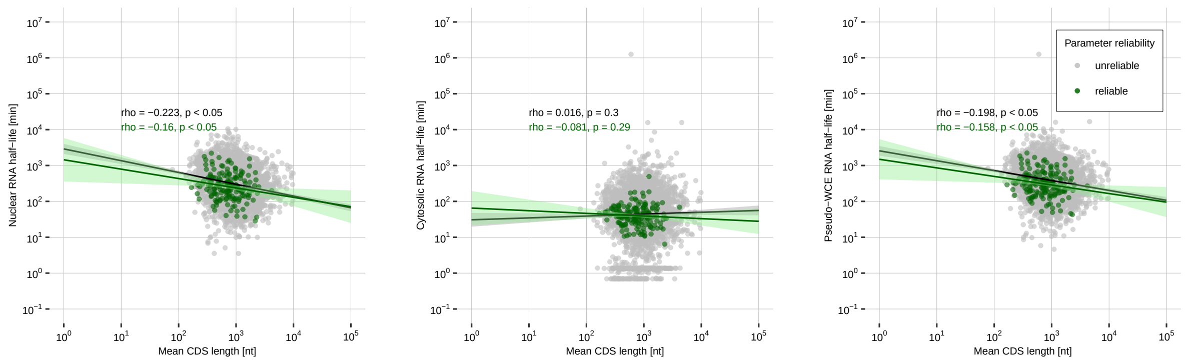

Fig N. **Scatterplot comparing the half-life estimates of our two-compartment model with the corresponding CDS length of a respective gene.**

5 Difference in RNA half-lives between 3'UTR isoforms

We used peak calling to define potential 3'UTR isoforms in an intragenic region. To that end, we defined peaks located in the same 3'UTR but separated by at least 100 nucleotides to be different 3'UTR isoforms. Differences in nuclear respectively cytosolic half-lives between these isoforms are shown in Fig O-Fig P.

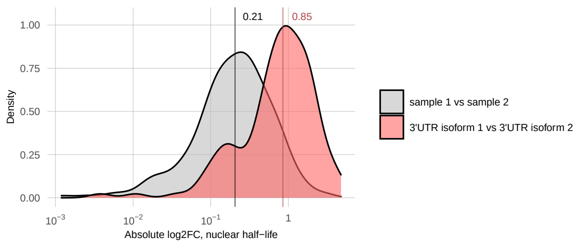

Fig O. **Difference in nuclear half-lives of potential 3'UTR isoforms.** Some 3'UTRs harbored at least two expressed and distant peaks, representing potential 3'UTR isoforms (242 peaks from 118 3'UTRs). The absolute log fold change of both nuclear and cytosolic half lifes was computed for all pairwise comparisons of the potential isoform irrespective of the half life estimate reliability. As comparison, absolute log2 fold changes were calculated for an isoform's estimates between time series 1 and 2. The medians are indicated by the red and black lines with corresponding text labels.

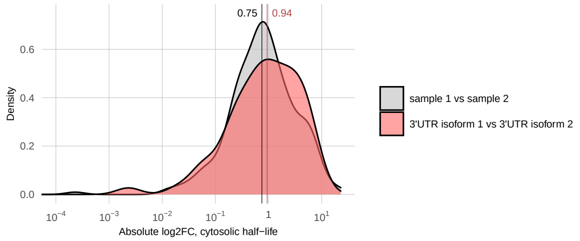

Fig P. **Difference in cytosolic half-lives of potential 3'UTR isoforms.** Some 3'UTRs harbored at least two expressed and distant peaks, representing potential 3'UTR isoforms (242 peaks from 118 3'UTRs). The absolute log fold change of both nuclear and cytosolic half lifes was computed for all pairwise comparisons of the potential isoform irrespective of the half life estimate reliability. As comparison, absolute log2 fold changes were calculated for an isoform's estimates between time series 1 and 2. The medians are indicated by the red and black lines with corresponding text labels.

6 RBP analysis

Protein-RNA-binding information was obtained from an eCLIP experiment from the ENCODE project [7, 8]. The data set comprises 120 distinct RBP-RNA interaction profiles in K562 cells. We considered BED files for the hg19 genome assembly only, to match the genome assembly used in our SLAM-seq experiments. We used GENCODE annotation [9] for the mapping of protein binding sites to genes. Based on the fact that poly-adenylated mRNA barely contain any introns, we excluded intronic protein binding sites. To control the number of false positives, we only kept binding sites passing stringent cutoffs (-log10(p-value) ≥ 5 and log2(fold-enrichment) ≥ 3).

Considering only 3'UTRs with a reliable rate estimate, we define a 3'UTR to be bound by an RBP, if this region can be uniquely assigned to one gene, and this gene also contains a binding site for that RBP. This way we obtain, for each RBP, a set of bound and a set of unbound transcripts. A Wilcoxon rank sum test was applied to compare if the nuclear or cytosolic half-life distribution of the bound transcripts differs significantly from the one of the unbound transcripts. The p-values were Bonferroni corrected for multiple testing.

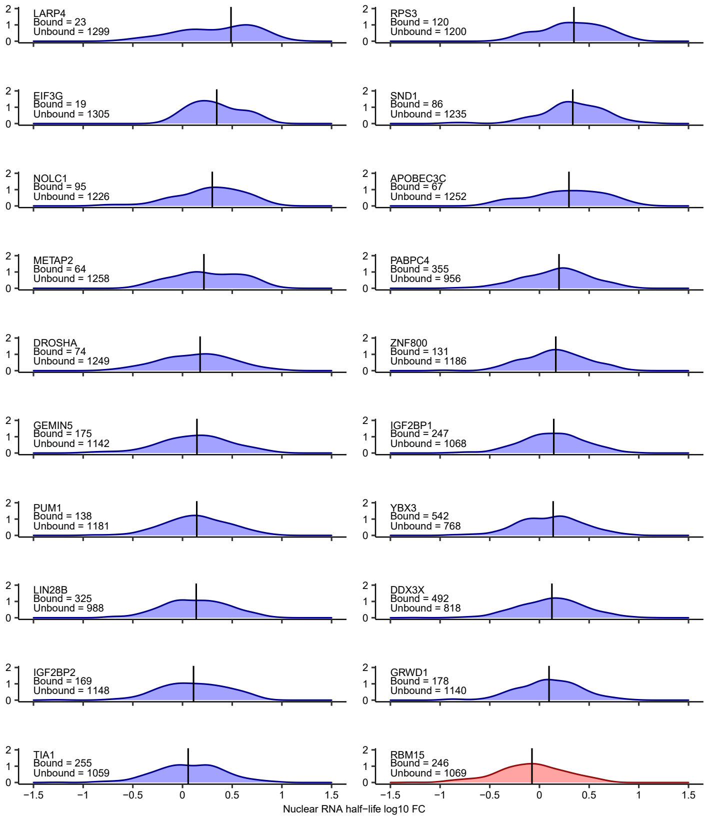

Fig Q. **Density plots for distinct RBPs from the eCLIP analysis (nuclear RNA half-lives).** Shown are the distributions of the nuclear half-lives from RNAs putatively bound to the respective RBP according to eCLIP experiments. Log10 fold-changes were calculated by taking the log10 of the nuclear half-lives and substracting the log10 of the global median (e.g. the median of all bound and unbound transcript half-lives) from these values. Blue densities indicate distributions with a higher median half live of the bound transcripts as the global median. The red density indicates a shorter median transcript half-life of the bound RNAs in comparison with the global median. Black bars indicate the median of the respective distribution.

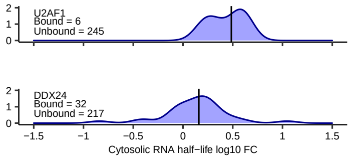

Fig R. **Density plots for distinct RBPs from the eCLIP analysis (cytosolic RNA half-lives).** Shown are the distributions of the cytosolic half-lives from RNAs putatively bound to the respective RBP according to eCLIP experiments. Log10 fold-changes were calculated by taking the log10 of the cytosolic half-lives and substracting the log10 of the global median (e.g. the median of all bound and unbound transcript half-lives) from these values. Blue densities indicate distributions with a higher median half live of the bound transcripts as the global median. The red density indicates a shorter median transcript half-life of the bound RNAs in comparison with the global median. Black bars indicate the median of the respective distribution.

7 Assessment of half-lives of lncRNAs and mRNAs translated at the ER

A 3'UTR was considered part of a lncRNA if it was assigned to exactly one, lncRNA gene. A collection of genes whose transcripts are likely translated at the ER was assembled from the following 6 GO terms: GOCC_INTEGRAL_COMPONENT_OF_CYTOPLASMIC_SIDE_OF_ENDOPLASMIC_RETICULUM_MEMBRANE, GOCC_INTRINSIC_COMPONENT_OF_ENDOPLASMIC_RETICULUM_MEMBRANE, GOCC_LUMENAL_SIDE_OF_EN[illegible] GOCC_PERINUCLEAR_ENDOPLASMIC_RETICULUM, GOCC_ROUGH_ENDOPLASMIC_RETICULUM, GOCC_ROUGH_ENDOPLASMIC_RETICULUM_MEMBRANE. A 3'UTR was considered part of a likely ER-translated transcript, if it was assigned to exactly one gene, and this gene was part of either these GO terms.

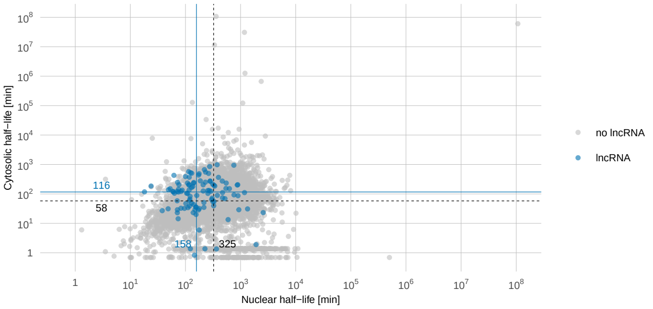

Fig S. **Half-life estimates of lncRNAs in comparison to all 3'UTRs.** From all expressed 3'UTRs, some were annotated as lncRNAs (blue data points). The blue lines indicate the median half-lives of the lncRNAs, the black lines indicate the median half-lives of all expressed 3'UTRs, respectively.

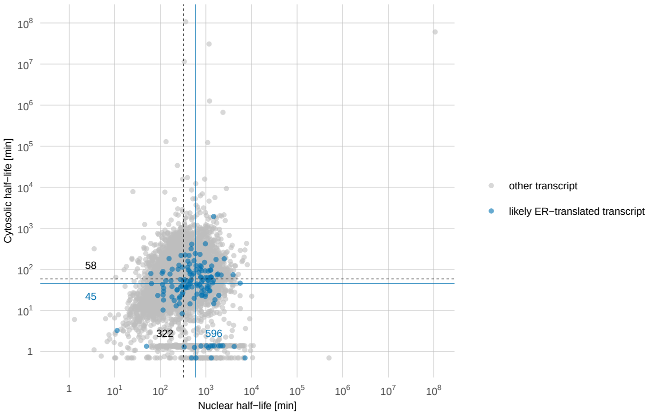

Fig T. **Median half lifes of transcripts likely translated at the ER.** From all expressed 3'UTRs, two subsets of transcripts were selected which are suspicious of being translated at the ER using appropriate GO. The blue and black lines with corresponding text labels indicate the median half lifes of the 3'UTR subsets and all expressed 3'UTRs, respectively.

8 Model including nuclear retention

In comparison to our standard two-compartment model (see main text equations (1) and (2)), we split the nuclear RNA N into two fractions, namely the fraction E that will be exported, and the retained fraction R, $N = E + R$. We assume that the decision whether an RNA is exported or not is made co-transcriptionally. Hence we introduce an additional retention paramter $r \in [0, 1]$ which determines the probability by which a transcript will be retained after completion of the transcription process.This leads to the ODE system

$$\dot{E} = \mu(1 - r) - (\nu + \tau)E \tag{3}$$

$$\dot{C} = \tau E - \lambda C \tag{4}$$

$$\dot{R} = \mu r - \nu R \tag{5}$$

with the boundary conditions $R_{new}(0) = E_{new}(0) = C_{new}(0) = 0$. When cells are in dynamic steady state, we have $R_\infty = \frac{\mu r}{\nu}$, $E_\infty = \frac{\mu(1-r)}{\nu+\tau}$, $C_\infty = E_\infty \frac{\tau}{\lambda}$. The ODE for old RNA equal those for new, with the exception that the terms including μ are omitted (or, μ is set to 0). This leads to the closed form solutions

$$R_{old}(t) = R_\infty \cdot e^{-\nu t} \tag{6}$$

$$E_{old}(t) = E_\infty \cdot e^{-(\nu+\tau)t} \tag{7}$$

$$C_{old}(t) = C_\infty \cdot (\frac{\lambda e^{-(\nu+\tau)t} - (\nu+\tau)e^{-\lambda t}}{\lambda - (\nu+\tau)}) \tag{8}$$

Correspondingly, the new fraction time curves are obtained by subtracting the old RNA levels from the steady state levels:

$$R_{new}(t) = R_\infty \cdot (1 - e^{-\nu t}) \tag{9}$$

$$E_{new}(t) = E_\infty \cdot (1 - e^{-(\nu+\tau)t}) \tag{10}$$

$$C_{new}(t) = C_\infty \cdot \left(1 - \frac{\lambda e^{-(\nu+\tau)t} - (\nu+\tau)e^{-\lambda t}}{\lambda - (\nu+\tau)}\right) \tag{11}$$

Subsequently, we get nuclear, respectively cytsolic new/total ratios for each timepoint t by:

$$\frac{N_{new}(t)}{N_\infty} = \frac{R_{new}(t) + E_{new}(t)}{R_\infty + E_\infty} = \frac{\frac{r}{\nu} \cdot (1 - e^{\nu t}) + \frac{1-r}{\nu+\tau} \cdot (1 - e^{-(\nu+\tau)t})}{\frac{r}{\nu} + \frac{1-r}{\nu+\tau}} \tag{12}$$

$$\frac{C_{new}(t)}{C_\infty} = 1 - \frac{\lambda e^{-(\nu+\tau)t} - (\nu+\tau)e^{-\lambda t}}{\lambda - (\nu+\tau)} \tag{13}$$

Given the observed nuclear and cytosolic new/total RNA ratios, Equations (12), (13) are then used to fit the parameters τ, λ, r, for a grid of values ν. We varied the value for the nuclear degradation rate ν extensively, to ensure realistic levels of accumulation of the retained RNA fraction. We used a three-dimensional MCMC to obtain a representative sample and an optimized fit for the nuclear export rate τ,the nuclear retention rate r and the cytosolic degradation rate λ. The sampler was initialized with the results of a global optimization algorithm (Differential Evolution algorithm, R command DEoptim). Next, the component-wise medians of the MCMC samples were computed after burn-in. These medians were used for another global optimization (Differential Evolution algorithm) to obtain the final parameter estimates for τ, r and λ. Parameter fitting was seperaterly perforemd for both samples, and estimates averaged afterwards. Further, we calculate the

percentage of retained transcripts as

$$\frac{R_\infty}{R_\infty + N_\infty} = \frac{\tau r + \nu r}{\tau r + \nu} \tag{14}$$

9 One-compartment model of the cytosolic RNA

A one-compartment model of the cytosolic compartment is given by

$$\dot{C}(t) = \mu - \lambda C \tag{15}$$

with boundary conditions $C_{new}(0) = 0$ and $C_{old}(0) = C_\infty = \frac{\mu}{\lambda}$. Fitting of λ was performed as described in Material and Methods for the nuclear compartment , separately for the two time series, and estimates were averaged afterwards.

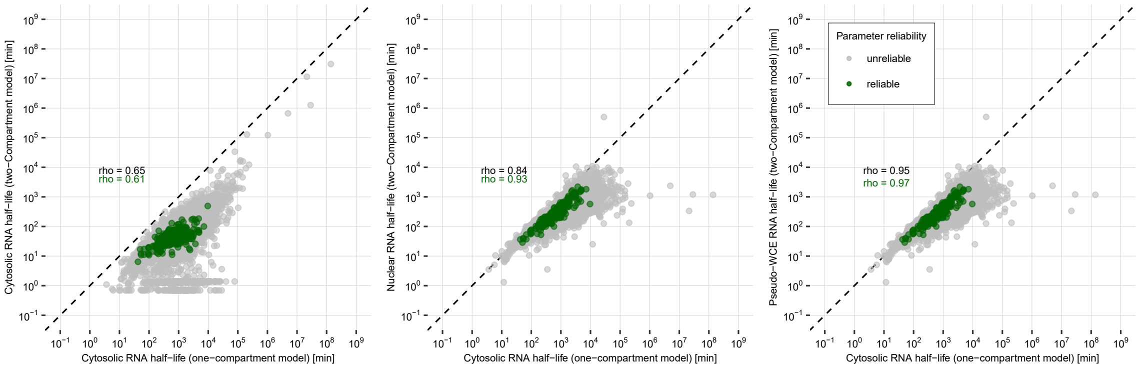

Fig U. **Comparison of estimates derived from a two-compartment and one-comparmtent model.** For visualisation purposes, the plot was cropped leaving out 2 data points.

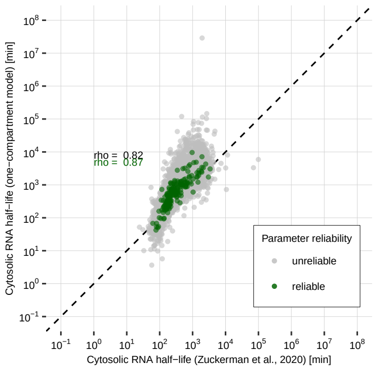

Fig V. **Correlation of a simple exponential cytosolic decay model fit results with cytosilc half-lives obtained through SLAM-seq on the cytosolic fraction of MCF7 cells by Zuckerman et al. (2020) [3].** Gray dots represent expressed 3'UTRs and green dots portray 3'UTRs that passed our reliability criteria for the cytosolic compartment.

References

[1] Schueler M, Munschauer M, Gregersen LH, Finzel A, Loewer A, Chen W, et al. Differential protein occupancy profiling of the mRNA transcriptome. Genome Biology. 2014 Jan;15(1):R15. Available from: https://doi.org/10.1186/gb-2014-15-1-r15.

[2] Wu Q, Medina SG, Kushawah G, DeVore ML, Castellano LA, Hand JM, et al. Translation affects mRNA stability in a codon-dependent manner in human cells. eLife. 2019 apr;8:e45396. Available from: https://doi.org/10.7554/eLife.45396.

[3] Zuckerman B, Ron M, Mikl M, Segal E, Ulitsky I. Gene Architecture and Sequence Composition Underpin Selective Dependency of Nuclear Export of Long RNAs on NXF1 and the TREX Complex. Molecular Cell. 2020 Jul;79(2):251-67.e6. Available from: https://www.sciencedirect.com/science/article/pii/S1097276520303142.

[4] Weiszfeld E, Plastria F. On the point for which the sum of the distances to n given points is minimum. Annals of Operations Research. 2009 Mar;167(1):7-41. Available from: https://doi.org/10.1007/s10479-008-0352-z.

[5] Hartley R, Aftab K, Trumpf J. L1 rotation averaging using the Weiszfeld algorithm. In: CVPR 2011; 2011. p. 3041-8. ISSN: 1063-6919.

[6] Fletcher PT, Venkatasubramanian S, Joshi S. The geometric median on Riemannian manifolds with application to robust atlas estimation. NeuroImage. 2009 Mar;45(1, Supplement 1):S143-52. Available from: https://www.sciencedirect.com/science/article/pii/S1053811908012019.

[7] Luo Y, Hitz BC, Gabdank I, Hilton JA, Kagda MS, Lam B, et al. New developments on the Encyclopedia of DNA Elements (ENCODE) data portal. Nucleic Acids Research. 2020 Jan;48(D1):D882-9. Available from: https://doi.org/10.1093/nar/gkz1062.

[8] Van Nostrand EL, Freese P, Pratt GA, Wang X, Wei X, Xiao R, et al. A large-scale binding and functional map of human RNA-binding proteins. Nature. 2020 Jul;583(7818):711-9. Number: 7818 Publisher: Nature Publishing Group. Available from: https://www.nature.com/articles/s41586-020-2077-3.

[9] Frankish A, Diekhans M, Jungreis I, Lagarde J, Loveland J, Mudge JM, et al. GENCODE 2021. Nucleic Acids Research. 2021 Jan;49(D1):D916-23. Available from: https://doi.org/10.1093/nar/gkaa1087.
