# Supplementary material for: Nuclear export is a limiting factor in eukaryotic mRNA metabolism
Source: PLoS Comput Biol. 2024 May 16;20(5):e1012059. doi: 10.1371/journal.pcbi.1012059 (PMC11135743; doi:10.1371/journal.pcbi.1012059)
Supplement: Attachment — Submitted filename: PLOS_responseletter_for_Reviewer.pdf [file pcbi.1012059.s006.pdf]

Responses to the reviewers' comments

For your convenience, the original comments are reproduced in italics, followed by our responses, which are indented.

Reviewer #1

In this study, Mueller et al. performed SLAM-seq time series experiments that are based on metabolic labeling using the nucleotide analog 4-thiouridine (4sU). Following metabolic labeling cells were fractionated into nuclear and cytosolic fractions, and the nuclear and cytoplasmic RNAs are subjected to SLAM-seq library preparation and 3'-RNA-seq, obtaining subcellular resolution. The authors use the time course SLAM-seq data to develop a two-compartment model of RNA metabolism to quantify the key RNA metabolic parameters: the mRNA synthesis-, nuclear export-, nuclear- and cytosolic degradation-rates. The authors find that degradation of polyadenylated mRNA in the nucleus is negligible and that nuclear export is slow whereas the degradation of mRNA in the cytosol is relatively fast. From these findings the authors conclude that polyA(+) mRNAs generally spend most of their time in the cell nucleus and that RNA export is a rate limiting factor in the mRNA's life cycle. The authors also provide evidence that the nuclear export rate can differ strongly for transcript isoforms with different 3'-UTRs suggesting that the 3'-UTR is an important determinant for nuclear export rates. Interestingly, the authors find that a subset of genes are activated upon metabolic labeling. This set of labeling-induced mRNAs exhibit higher nuclear export rates as other mRNAs which points to different mRNA export pathways.

The findings of this combined experimental and computational study are exciting. Of particular relevance, at least in my opinion, is the finding that polyadenylated mRNAs generally spend most of their life time in the nucleus. The methods that the authors have used are state-of-the-art. The experiments were well designed and the advanced computational analyses have been thoroughly performed. The study opens new questions that can be addressed in future studies. For instance, the data suggests the co-existence of alternative export pathways. The identification and characterization of these pathways represents an important future direction. The findings of this work will be of broad relevance for the large community of researchers with an interest in a systems-level understanding of the RNA life cycle and especially to those who work on mRNA metabolism and RNA export.

> We thank Reviewer #1 for her/his concise summary and appreciation of the potential impact of our work. Her/his comments helped us to be more precise in our terminology, which greatly improved the quality of our work.

Major concerns:

(1) The introduction section of the manuscript should be better adapted to the main subject and results of this study. The authors should also emphasize what is not known helping the reader to understand the motivation and need of this systems-wide RNA analysis.

> In the introduction, we now put more emphasis on the combination of subcellular fractionation and statistical modeling, which has not been performed before. E.g., RNA dynamics have not been studied across compartments. We also discuss the new challenges arising from using a two-compartment model instead of a one-compartment model.

(2) The first paragraphs of the results section (pages 3 to 7) are very technical and appear as part of the methods section. I understand that the authors present a new model to quantify main parameters of the RNA life cycle and therefore technical aspects are important. However, I find especially the first paragraphs of the results section hard to read. The authors may also want to consider to reduce the load of formulas in the main text.

Both paragraphs have been substantially revised as a result of this comment. Equations (1) and (2) introduce the two-compartment model and are indispensable. Former Equation (3) has been incorporated into the text to improve the flow of the text. The notation in Equations (4) and (5) has been further simplified. However, we retain both equations as they are essential for parameter learning. In short, they describe the time course of the relative abundances of labeled RNA with only two parameters (cytosolic degradation rate λ, nuclear removal rate ν+τ). Compared to the original four parameters of the two-compartment model, this greatly simplifies the task of parameter fitting and the risk of overfitting. Equation (6) has been moved to the Supplements.

(3) On page 3 the authors mention that two replicate samples were obtained per time point. The authors should provide a correlation heatmap with the correlation coefficients between the replicates and between time points. This would further (in addition to Fig. 1D) demonstrate the reproducibility of the data.

We show a scatter plot comparing the nuclear half-live estimates derived from the two independent time series in Supplemental Figure S18. The same is done for the cytosolic half-lives in Supplemental Figure S19. In both cases, the estimates correlate well (Spearman correlation $\rho = 0.9$ for the nuclear half-lives, $\rho = 0.6$ for the cytosolic half-lives). We now have added a comparison of the new/total ratio estimates between time points and between the two replicates as a correlation heat map in Supplemental Figure S17. As expected, the between-replicates agreement is high for identical time points (Pearson correlation $\rho > 0.84$ for each pairwise comparison). This demonstrates reproducibility and accuracy of our framework. Note that the correlation between time points decreases with duration of the interval between the time points. This is to be expected, as the new/total ratio increases constantly with time.

Minor concerns:

(1) The authors should mention the model system in the abstract: human cancer cells.

We now explicitly mention the model system (human cancer cells) in the abstract.

(2) A more detailed description of how 3'-UTR isoforms were defined in the methods section would be helpful. How many 3'-UTR isoforms were identified? How many per transcript?

We now have better motivated why we perform read aggregation at the 3'UTR level in first place. The characterisation of 3'UTRs is given in the first Results section (Experiments and primary data processing). Here, we also provide details on how the 3'UTRs were defined and how many of them could be quantified in our experiments. For clarity, we have renamed this paragraph "SLAM-seq time-series and 3'UTR quantification", and we have modified the text.

(3) The authors nicely compare their obtained RNA half-lives (HeLa) with already available half-lives from MCF7 and HEK293 obtained in previous studies by others. The authors found that the half-live estimates from HEK293 and MCF7 cells were systematically shorter or longer than the half-lives obtained for HeLa cells (Page 7). What are potential reasons for the systematic differences? The authors could elaborate on this in the main text.

We thank Reviewer #1 for pointing this out. Different cell lines indeed have differences in proliferation rates and also in RNA metabolism. The latter is clearly observed when we use metabolic labelling to isolate newly synthesised RNA, where the yield of newly synthesised RNA strongly varies by cell type when the same metabolic labelling time is used (K. Zumer, unpublished). We elaborate on these differences in the Discussion.

(4) Fig. 3B: text of the x-axis is partially overlapping

We have increased the distance between x-axis title and ticks in the plot to improve its visualisation.

Reviewer 2

Muller et al. presented a dynamic model of time-resolved SLAM-seq data to quantify two key parameters of RNA metabolism, namely the export of mRNA from nuclear compartment to cytosol and degradation. It is a two-compartment model, which should capture the main steps of RNA metabolism (mRNA synthesis, nuclear export, and nuclear and cytosolic degradation rates) more accurately than existing models like GRAND-SLAM that assume constant RNA synthesis rates, degradation rates, and a dynamic equilibrium of each RNA population.
The manuscript's main finding is that mRNA residence time in the nucleus is much longer than that in the cytosol. The authors also showed that different 3'UTR transcript isoforms can be exported at substantially different rates and that stress by 4sU labeling can induce alternative export pathways.

Overall, it is a well written paper with careful pre-processing of SLAM-seq data.

We thank Reviewer #2 for the thorough review and the positive evaluation of our manuscript.

Major concerns:

1. The authors analyzed one dataset, but consistently made general biological statements. As presented, it is unclear why the authors did not use other published datasets (e.g., https://elifesciences.org/articles/45396) and if their statements will generally hold on to other datasets.

We thank Reviewer #2 for drawing our attention to the abovementioned dataset. At the time of writing, we have performed thorough literature search for other suitable datasets that perform subcellular fractionation combined with RNA labeling. However, the only suitable study was the one by Zuckerman et al. (2020), which we included in our analyses. For further validation, we have added a comparison of the dataset suggested by Reviewer #2. This can now be found in the Supplements (Supplemental Figure S23). For convenience, we also

present it below. As can be seen, the results are similar to those we found for Zuckerman et al. (compare Supplemental Figure S28).

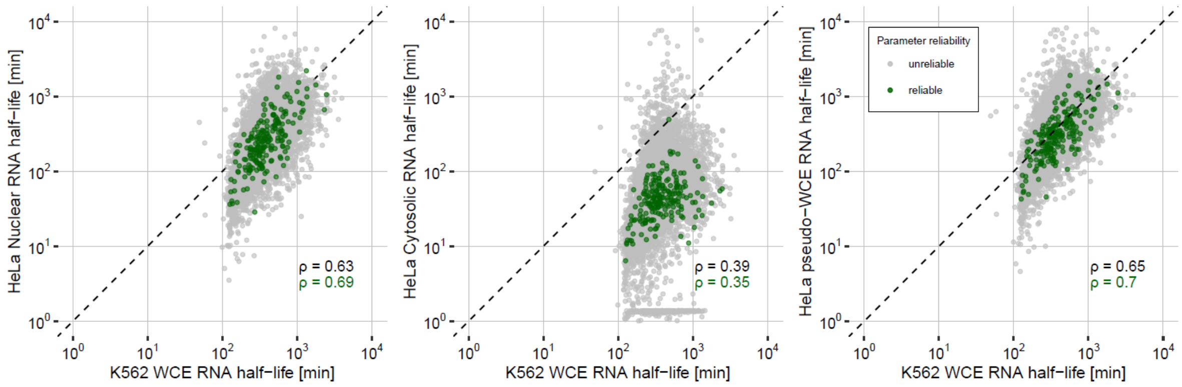

S23 Fig: Comparison between RNA half-lives from a two-copartment model and literature estimates obtained by a one-compartment model fit to SLAM-seq whole-cell extracts. Comparison between RNA half-life estimates by our two-compartment model and whole-cell extract half-life measurements from Wu et al. (2019). The nuclear and cytosolic RNA estimates were summed to generate pseudo-whole-cell estimates. Gray dots represent unreliable estimates, green points represent reliable estimates.

In the revised manuscript, we tried to avoid claims and broad generalizations which are unbacked by our data. During the revision phase, we have re-iterated our literature search for datasets that perform subcellular fractionation and RNA labelling. However, there was no dataset available.

2. The authors clearly mentioned their assumptions but didn't justify them well. For example, they noted: "Since our experiments are conducted under constant, optimal growth conditions, we assume these four rates are constant over time" and "we further assume that the metabolic parameters of 4sU-labeled RNA are identical to those of unlabeled RNA." Both assumptions are questionable unless presented with prior evidence. It is also critical to assess (at least, to comment on) how these assumptions might affect their conclusions.

We share the concerns of the reviewer. For this reason, we have performed extensive quality checks, and we have expanded our discussion on the (non-)toxicity of 4sU labeling at different concentrations.

- Labeling concentrations in the range of 100-1000 µM are common (Duffy, Schofield et al., 2018), without substantial effects on the transcriptome. Nonetheless, we are aware that 4sU labeling at higher concentrations (>100 µM) and longer labelling periods (>2h) can elicit cellular stress responses (Burger et al., 2013). However, Herzog et al. (2017) demonstrated similar growth under control and 4sU labeling conditions. For the reviewer's convenience, we attach the Figure from Herzog et al. (2017) below. Therefore, 4sU labelling is considered to introduce only minimal perturbations to the cell when grown under optimal conditions like in our case. We have added the above references and elaborate on 4sU labeling in the Introduction and the Methods section "Cell culture and subcellular fractionation".

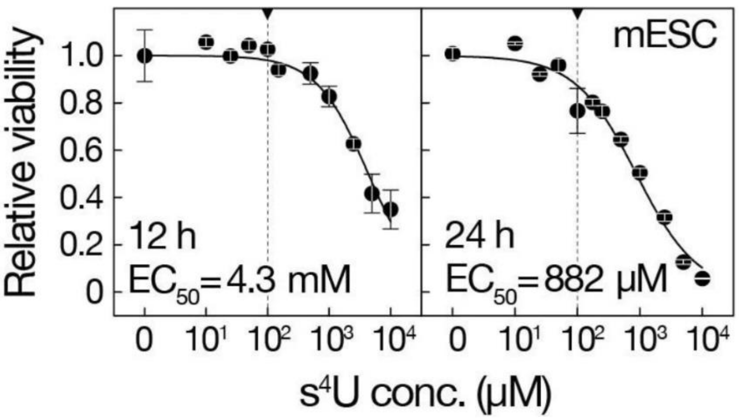

Figure 1 (taken from https://www.nature.com/articles/nmeth.4435/figures/10). Viability of mESCs cultured in the presence of the indicated concentration of 4-thiouridine (s^4U) for 12 h (left) or 24 h (right) relative to untreated conditions is shown. Note, that s^4U-containing media was exchanged every 3h in the course of the labeling experiment. Final concentration used in subsequent experiments (100 µM) is indicated by triangle and dotted line. Mean ± SD of three independent cell cultures are shown.

- We have added a comparison of total mRNA expression along the time course which did not find relevant effects of 4sU labeling on transcription (Supplementary Figure S16).

However, we were aware that 4sU labeling can substantially affect the expression of a few, individual transcripts. We show such an example (SGK1) in Fig 3F. Our bioinformatics protocol contains a priori and post-hoc quality filtering criteria that ensure we will only analyse and report the dynamic parameters of transcripts that adhere to our model assumptions and lead to reproducible estimates (see Methods “Reliability Criteria”). Note that our conclusions are based only on the “reliable” transcripts that meet all these criteria.

3. The assumption of global parameters for mRNA synthesis, export, and degradation is also questionable. Won’t the size of the dataset allow for at least binary categorizations (high/low and fast/slow) of these parameters?

To avoid misunderstanding, we would like to point out that the only global parameter (a parameter that is shared for all transcripts) is the labeling efficiency. We use this quantity to estimate transcript-specific rates of RNA metabolism, namely the synthesis rate μ, the nuclear export rate τ, and the nuclear and cytosolic degradation rates ν respectively λ. For simplicity, we have left out transcript-specific indices in the main text. We have changed this paragraph in the Results section (see "A two-compartment model of RNA metabolism") to clarify this.

4. Critical parts of the approach, such as estimation of new/total mRNA ratios, need to be validated on additional datasets. The authors seemed forceful at some places. For example, they noted in S5 that “Contrary to our intuition, the estimated value for the maturation gap was negative. We still keep our model, since it represents an excellent fit of labeling efficiency.”

We appreciate the in-depth assessment of our estimation procedure by Reviewer #2. While we share the desire to validate the new/total estimation approach on other datasets, there is no such data available. An ideal validation set would be an in vitro mixture of labelled and unlabelled RNA at defined, yet transcript-specific ratios. Additionally, the labelled transcripts should be labelled with a similar efficiency as observed in the in vivo experiments. A controlled

synthesis of such RNA would be extremely expensive, laborious and has never been tried so far.
We also value the careful reading of the supplements concerning the time-dependency of the labeling efficiency. We regret to have chosen the misleading term “maturation gap” for a quantity that might be more accurately denoted as “detection gap”. It describes a temporal offset (Δ) in the mathematical model of our saturation kinetic for 4sU incorporation. We discussed that such an offset could be due to co- and post-transcriptional RNA processing, hence the term maturation gap, and hence the confusion that such an offset cannot be negative as estimated from the data. However, other factors such as variations in the duration of labeling periods or delays in cell harvesting/processing can add up to an error of minutes and shift the offset in a negative direction. We have therefore changed the labeling of the term Δ to "detection gap" and added a discussion of the origin of the offset.
It is important to note that Δ does not play any role in the estimation of the RNA metabolic parameters, nor in the estimation of the labeling efficiency. The saturation model merely served the purpose of simulating a realistic saturation kinetic, which in turn was used to assess the estimation bias introduced by the effect that transcripts of the same RNA population were not labelled with the same efficiency (see Supplements “Modeling of the labeling efficiency in the nucleus and the detection gap”). Luckily, this effect turned out to be very subtle, and we do not elaborate on it in detail in the main text.

5. Due to points 2-4, much of the presented results remain questionable.

We did our best to clarify for point 2-4.

6. The authors wrote a very nice Introduction section, but similar elaborations of the SLAM-seq technology would be helpful.

We fully agree and have added more details on the SLAM-seq technology in the Introduction.

7. The github link (https://github.com/IMSBCompBio/mRNAdynamics) was not accessible.

We apologise for this inconvenience. Originally, we stated that for intellectual property reasons, reviewers could be granted access during the publication process upon request. Unfortunately, GitHub does not provide tokens for reviewers. We are happy to provide access during the review process, but please note that this may compromise the anonymity of the reviewers.
